# Raman spectroscopy for viral diagnostics

**DOI:** 10.1007/s12551-023-01059-4

**Published:** 2023-04-10

**Authors:** Jijo Lukose, Ajaya Kumar Barik, Mithun N, Sanoop Pavithran M, Sajan D. George, V. M. Murukeshan, Santhosh Chidangil

**Affiliations:** 1 Centre of Excellence for Biophotonics, Department of Atomic and Molecular Physics, Manipal Academy of Higher Education, 576104 Manipal, India; 2 Centre for Applied Nanosciences, Department of Atomic and Molecular Physics, Manipal Academy of Higher Education, 576104 Manipal, India; 3 Centre for Optical and Laser Engineering, School of Mechanical and Aerospace Engineering, Nanyang Technological University, 50 Nanyang Avenue, 639798 Singapore, Singapore

**Keywords:** Virus, Raman spectroscopy, Surface-enhanced Raman spectroscopy, Tip-enhanced Raman spectroscopy, Raman tweezer, SARS-CoV-2, Point of care applications

## Abstract

Raman spectroscopy offers the potential for fingerprinting biological molecules at ultra-low concentration and therefore has potential for the detection of viruses. Here we review various Raman techniques employed for the investigation of viruses. Different Raman techniques are discussed including conventional Raman spectroscopy, surface-enhanced Raman spectroscopy, Raman tweezer, tip-enhanced Raman Spectroscopy, and coherent anti-Stokes Raman scattering. Surface-enhanced Raman scattering can play an essential role in viral detection by multiplexing nanotechnology, microfluidics, and machine learning for ensuring spectral reproducibility and efficient workflow in sample processing and detection. The application of these techniques to diagnose the SARS-CoV-2 virus is also reviewed.

**Graphical abstract:**

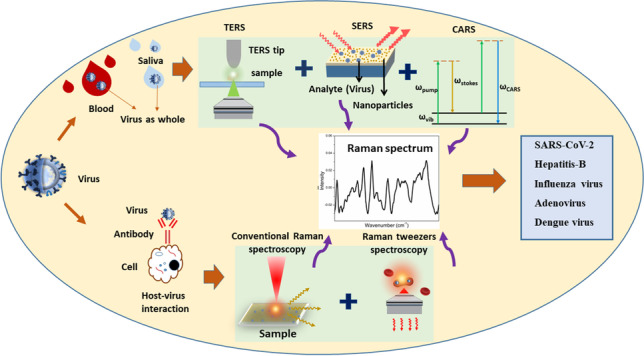

**Supplementary Information:**

The online version contains supplementary material available at 10.1007/s12551-023-01059-4.

## Introduction

Viruses, described as “organisms at the edge of life,” are known to be the causative agents of most of the human pandemics reported in history (Rybicki 1990). Viruses are infectious agents with submicroscopic sizes which are obligate intercellular parasites. The traditional methods of viral detection rely on viral isolation and culture techniques such as plaque assay, hemagglutination assay, and histological observations (Hematian et al. 2016; Storch 2000). However, these techniques demand skilled labor, a culture-based approach, and longer duration. Direct examination techniques such as electron microscopy or immunofluorescence usually suffer from poor sensitivity and specificity and the data can be difficult to interpret (Burrell et al. 2017). Polymerase chain reaction (PCR) techniques have been employed since the 1990s and represent the current gold standard (Espy et al. 2006). However, the high workload and reagent shortage during the epidemic makes testing during viral pandemics using the PCR technique a challenging task.

Spectroscopic modalities, Raman spectroscopy in particular, allow for detailed investigation of various kinds of biological samples, with one prominent example being cancer detection (Ibrahim et al. 2021). The use of Raman spectroscopy for viral detection from body fluids has seen a resurgence (Saleem et al. 2013; Saade et al. 2008; Naseer et al. 2019; Desai et al. 2020). Virus detection can be achieved either directly, by identifying the components of the virus, or indirectly by detecting components from the virus elicited immune response (Ramoji et al. 2022). The standard target biomolecules in the former case are glycoproteins, nucleocapsid, and viral genome; the typical target biomolecules for the latter case are antibodies and cytokines (Yang et al. 2021). The change in relative distribution of various components such as proteins, amino acids, lipids, and carbohydrate derivatives in body fluids can also act as an indicator of viral infection. Capturing such biochemical signatures in the nanoscale regime can be realized by versatile Raman spectroscopic techniques such as surface-enhanced Raman spectroscopy (SERS), Raman tweezers, tip-enhanced Raman spectroscopy (TERS), and coherent anti-Stokes Raman scattering (CARS). Recently, Savinon-Flores et al. have reviewed SERS-based methods for the detection of viral infections in humans (Savinon-Flores et al. 2021). The present study extends this discussion to other variants of Raman spectroscopy.

## Raman spectroscopy

**Fig. 1:**
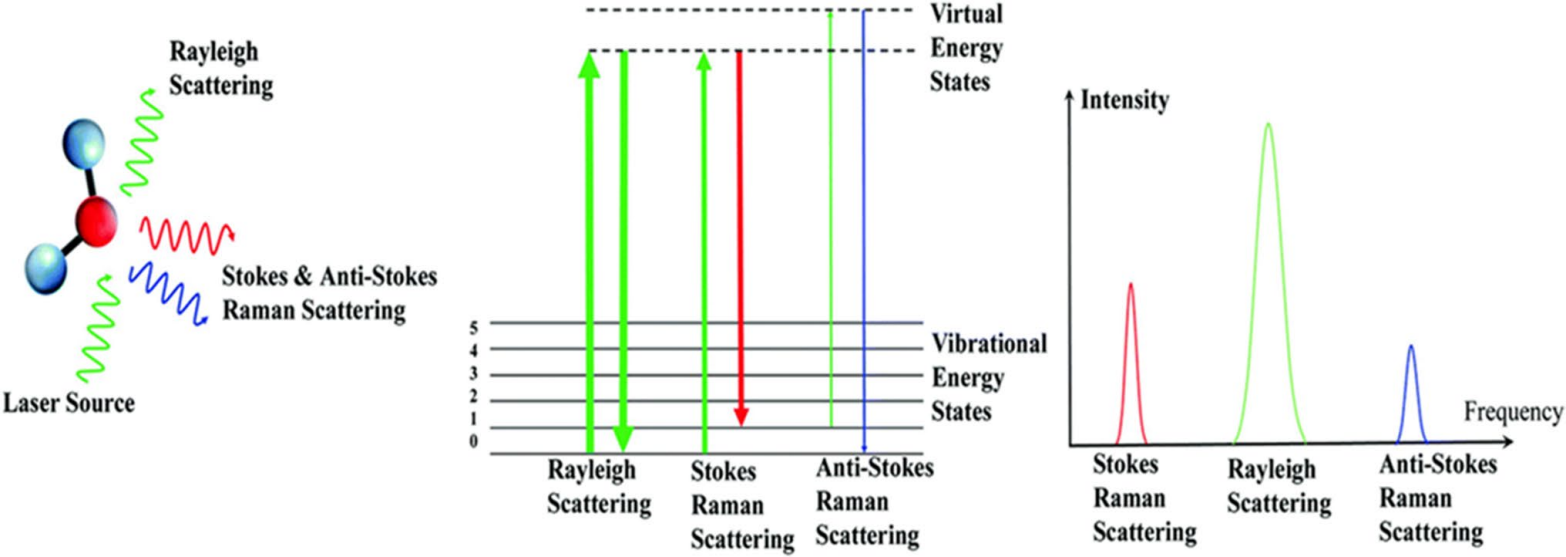
Conceptual representation of scattering process (George 2020)

Raman spectroscopy is a technique used for the determination of molecular structure based on vibrational frequencies of bonds by analyzing the light scattered from the sample of interest (Das and Agrawal 2011). This technique utilizes the change in polarizability of the molecular species upon irradiation with electromagnetic radiation and a concomitant inelastic scattering to fingerprint the chemical identity of the molecule. The inelastically scattered light (Raman scattering) produced by excitation with monochromatic radiation contains rich information about molecular vibrations which has led to it becoming a popular technique to analyze many kinds of biological samples (e.g., tissues and body fluids) (Dietzek et al. 2010). The efficiency of Raman scattering is typically very low, such that it accounts for only 1 in 10 million incident photons. In Stokes Raman scattering, the scattered radiation has lower energy compared to the incident radiation, whereas, in anti-Stokes Raman scattering, the scattered radiation has higher energy than that of the incident light (Popp and Kiefer 2006). The schematic of the Raman scattering process is shown in Fig. 1 (George 2020). The Stokes scattering is more favored as compared to the anti-Stokes scattering under ambient conditions. In Raman measurements, the dominant Rayleigh scattered radiation is filtered out to obtain the Raman signal from the sample of interest. The popularity of Raman spectroscopy for investigating biological samples can be attributed to the insignificant Raman scattering of the water, which is the major component in almost all biological entities. This technique does not require expensive solvents, matrix, sample processing steps, or lengthy data analysis time.

Advancements in optical instrumentation have facilitated the usage of the Raman technique in a wide range of biomedical applications. These developments have involved improvements in light sources and detectors relating to diode lasers, CCDs, mini-spectrometers, and optical fiber probes (Zhu et al. 2014). Nanotechnology-based developments in Raman spectroscopy has led to SERS, which has allowed for ultra-sensitive detection of biomolecular analytes when present at very low concentrations in body fluids (Culha et al. 2012).

## Raman spectroscopy in viral diagnostics

Biosensors exploiting the Raman effect have been reported for a variety of viruses, and a few are discussed here. Micro-Raman spectroscopy has been used to investigate adenovirus-infected human embryonic kidney epithelial (HEK293) cells (Moor et al. 2014). The study was capable of detecting viruses within cells from the spectra recorded after 12 h of viral infection, which is comparatively quicker than the routinely adopted immunostaining technique. The researchers identified one Raman spectral line at around 952 cm^−1^ that can be used as a spectral marker for identifying virus proliferation in cells. In 2018, the same group demonstrated the detection of adenovirus in the human cell within 3 h of viral infection (Moor et al. 2018). They suggested that two mechanisms occur in the cell following virus invasion. An initial reaction, proceeding without any protein production or nucleic acid replication, occurs immediately after viral entry, and a later phase in which both nucleic acid replication and viral protein production occur. Raman spectra were shown to be effective in identifying the compositional changes in DNA and RNA due to the cellular response upon viral invasion. In another work, the Raman technique was used to detect herpes virus in cell cultures (Salman et al. 2014). These experiments conducted on Vero cells were able to discriminate between control and herpes simplex virus type 1 (HSV-1) infected cases with 100% sensitivity. The major spectral contributions were exhibited in the amide III region comprising bands arising from proteins, lipids, and nucleic acids. Raman spectroscopy has been also employed for evaluating the biochemical changes induced due to Epstein–Barr virus infection in human glial cells (Tiwari et al. 2020). That study found differences in the Raman spectra obtained from the nucleus and periphery, which indicates that the viral invasion and its progression have resulted in different signaling processes at these locations. Combined with multivariate analysis, Raman techniques have been used to detect porcine parvovirus from cell cultures with a sensitivity and accuracy of 97.77% and 99.97%, respectively (Gogone et al. 2021).

Saleem et al. have applied the Raman technique to whole blood and serum samples for the detection of dengue viral infection. The experiments conducted using both 532 nm and 442 nm laser excitations were able to identify the dengue infection from the Raman marker bands at 1614 cm^−1^ and 1750 cm^−1^. These bands were originated from the presence of antibodies (IgG and IgM) in blood samples induced in response to viral infection (Rehman et al. 2012). Later, the same group employed partial least square (PLS) regression modeling on the Raman measurements of infected serum samples achieving an accuracy of 100% when tested on six blind samples (Saleem et al. 2013). Bands (at 750 and 850 cm^−1^) corresponding to adenosine diphosphate (ADP) that arose from thrombocyte cell rupture were observed in dengue virus-infected cases. Besides, the presence of cortisone signature at 1732 cm^−1^ and the absence of carotenoid bands (1006, 1156, and 1516 cm^−1^) were found in the case of infected cells. Saade et al. have used the Raman technique to probe hepatitis virus in blood samples (Saade et al. 2008) by observing variation in the phospholipid band intensities which they attributed to enzymes triggered by hepatocytes damage in hepatitis. Naseer and his team differentiated between dengue and typhoid infections in human serum by use of the Raman spectroscopy technique (Naseer et al. 2019). This study was successful in identifying specific Raman biomarkers for both bacterial and viral infections from the serum sample. A band at 1686 cm^−1^ was proposed as a marker for dengue viral infection, which originated from soluble ST2 protein in dengue patients. In a recent pilot study, Desai et al. used Raman spectral features for the identification of viral RNA from saliva, with a sensitivity of 7.05 × 10^7^ TU/mL (transduction units per mL) viral particles (Desai et al. 2020).

**Fig. 2:**
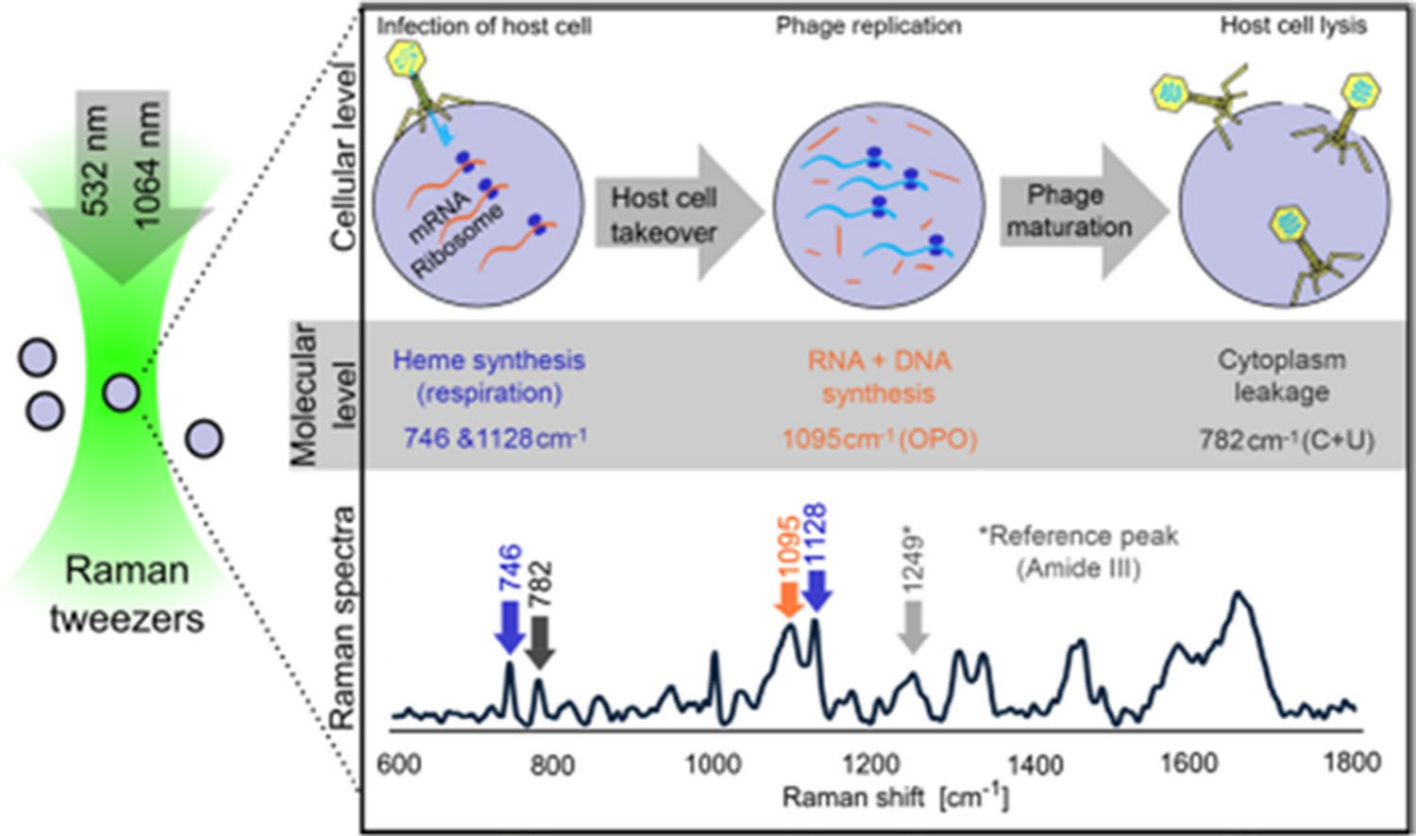
Bacteriophage invasion and corresponding molecular changes probed through Raman tweezer. Reprinted with permission from Pilat et al. (2020)

In a recent report, the automated deep learning-based TL-ResNet101 approach was employed to the analysis of Raman spectral images for identifying HBV infection from plasma (Ali et al. 2021). Deep transfer learning (DTL) was used to identify variations in infected Raman spectra and demonstrated high sensitivity (100%) as well as accuracy (99.7%) for hepatitis B virus (HBV) diagnosis. Another recent work used multiscale convolution neural networks (MCNN) for the classification of hepatitis infections from the Raman spectra of the serum sample (Zhao et al. 2021). Monsees et al. identified the Raman peak intensity ratio between nucleic acids and proteins useful for classification of virally infected cells from that of uninfected. They used Raman micro-spectroscopy for collecting the Raman signal from the phi6 (bacteriophage) infected *Pseudomonas syringae* and phi29 infected *Bacillus subtilis* as well as corresponding controls (Monsees et al. 2022). They observed an increase in the ratio for the former case and a decrease in the latter. Raman tweezers is a variant of Raman spectroscopy which is used in combination with optical tweezers to facilitate the investigation of live cells in physiological media without the requirement of any chemical/physical fixation. Raman tweezers use an optical trap (created by a tightly focused laser beam) to optically arrest the biological cell with simultaneous measurement of Raman spectra from the sample. This technique was used to study the influence of exogenous stress agents on human red blood cells. This includes monitoring the impact of extracellular tonicity, metallic nanoparticles, bisphenol A, alcohol, and normal saline on human red blood cells. We have previously demonstrated the use of Raman tweezers to investigate the response of single cells to external factors (Bankapur et al. 2010, 2014; Barkur et al. 2015; Lukose et al. 2019). Raman tweezers have also been used for the identification of Kaposi’s sarcoma-associated herpesvirus (KSHV) infected cells (BCBL-1 and BC-1 cells) from uninfected BJAB cells (Hamden et al. 2005). Raman tweezers were used to identify herpes viral infection based on Raman frequencies of proteins and nucleic acids at 1004, 1093, and 1664 cm^−1^ (Hamden et al. 2005). Pilat and team employed the Raman tweezer technique for analyzing the *Staphylococcus aureus* infected by two virulent phages JK2 and 80α, identifying band intensity at 746 cm^−1^ and at 1128 cm^−1^ attributed to heme required for energy production at the initial stage of infection (Pilat et al. 2020). Phage replication inside the host was shown by following Raman spectra intensity corresponding to the phosphodiester bond (1095 cm^−1^). As the host cells leak their cytoplasmic content during lysis, this leads to a decrease of cytosine and uracil peak at 782 cm^−1^ (Pilat et al. 2020). The corresponding Raman analysis scheme is shown in Fig. 2.

**Fig. 3:**
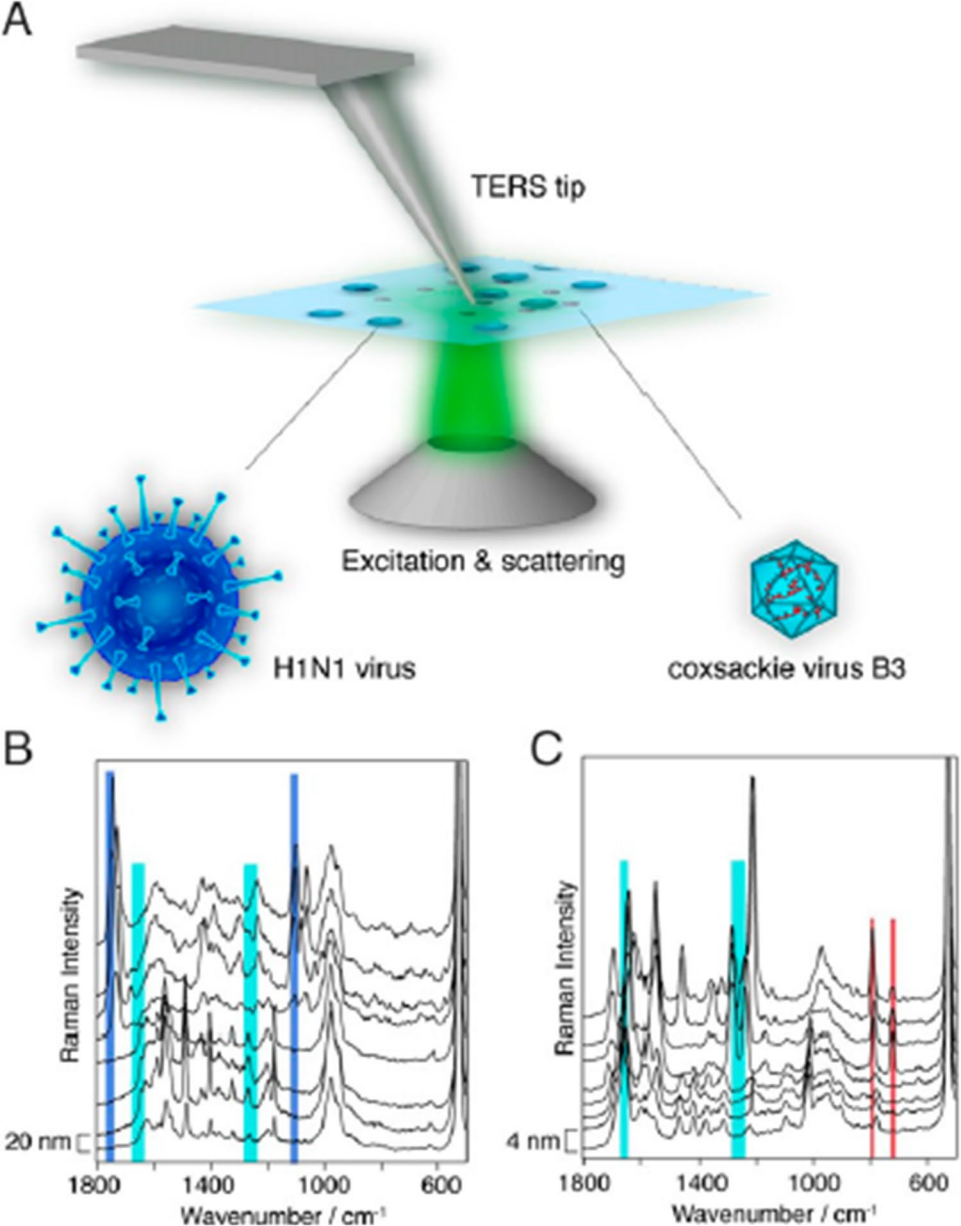
Schematics of identification of individual viruses (H1N1 and coxsackie virus) using TERS multiplexed with CARS. Reprinted from Deckert et al. (2020)

Cialla and colleagues demonstrated the applicability of TERS for viral investigations by extracting Raman signatures from the coat proteins and RNA of mosaic virus (Cialla et al. 2009). Ideally, the TERS technique combines the advantage of chemical sensitivity of the SERS technique with the high spatial resolution at the nanometer scale of a scanning probe technique. Nanoscale chemical imaging is realized in this technique with the aid of the electromagnetic field confined to the tip of a scanning probe microscope (SPM) (due to localized surface plasmon resonance) the so-called lightning-rod-effect (Cialla et al. 2009). The Abbe diffraction limit of the conventional Raman technique was shown to be circumvented by the single point TERS technique when used for investigating avipoxvirus (APV) and adeno-associated virus (AAV). Changes in the relative peak intensities as well as the position of spectral features were recorded for different particles from different virus strains (Hermann et al. 2011). In other recent work, a two-component approach employing TERS and AFM-IR techniques was used for both the identification as well as the structural characterization of individual virions (Dou et al. 2020). This combined approach was effective in determining protein secondary structure and amino acid composition of viral surfaces and was also able to probe the structural organization of individual virions of herpes simplex virus type 1 (HSV-1) and MS2 (Dou et al. 2020). Popp et al. used this technique together with chemometric analysis for the discrimination of varicella-zoster virus (VZV) and porcine teschovirus (PTV) with an accuracy of 91% (Olschewski et al. 2015). Single virus detection ability of this technique was demonstrated for classification of influenza virus and picornavirus (Deckert et al. 2020). The possibility of CARS technique for viral detection has been proposed, where the high electromagnetic field enhancement arising from the four-wave mixing of non-linear optics can overcome the limitations of weak inherent signals in the Raman technique (Deckert et al. 2020). The possibility of combining femtosecond adaptive spectroscopic techniques with enhanced resolution (FASTER)-CARS has been applied for individual viral particle detection (Deckert et al. 2020). Deckert et al. have achieved both quantitative and qualitative analyses at single virus level by effectively combining the tip-enhanced microscopy with coherent anti-Stokes Raman scattering (Fig. 3A). They were able to identify single copies of the H1N1 virus and coxsackie B3 virus solely based on the surface composition, as shown in Fig. 3B, C (Deckert et al. 2020). Detailed account of a single virus could be acquired since the tip is scanning the virus surface several times unlike the conventional SERS, where the averaging is done by investigating a large number of individual samples.

Resonance Raman spectroscopy (RRS), another variant of Raman spectroscopy, is a technique, where the incident laser energy is selected to be as close to the electronic transition energy of the sample (Butler et al. 2016). RRS has been effective in providing an enhancement factor of 10^2^–10^6^ for the Raman active vibrational frequency modes, which affords improvement of the detection limit (Butler et al. 2016; Wood and McNaughton 2006; Wood et al. 2004; Kuzmin et al. 2016). However, background fluorescence originating from the samples is a matter of concern in RRS. Use of UV-resonance Raman spectroscopy has been shown to be useful for analyzing virological samples, especially samples like filamentous viruses (Wen and Thomas 1998, Wen et al. 1999, 1997; Tadesse et al. 2020). Wen et al. have studied virus constituents using four different UV excitation wavelengths (257, 244, 238, and 229 nm) and were able to generate a databank for aromatic amino acids from proteins as well as for DNA nucleosides (Wen and Thomas 1998). Although identical signatures for coat protein tyrosine were found in filamentous viruses Pf1 and fd virions, the use of UV-resonance Raman spectroscopy indicated that the DNA organization was different (Wen et al. 1999). Supplementary Table 2 summarizes studies on detection of viruses of different kinds using Raman spectroscopy techniques.

## Surface-enhanced Raman spectroscopy for virus detection

**Fig. 4:**
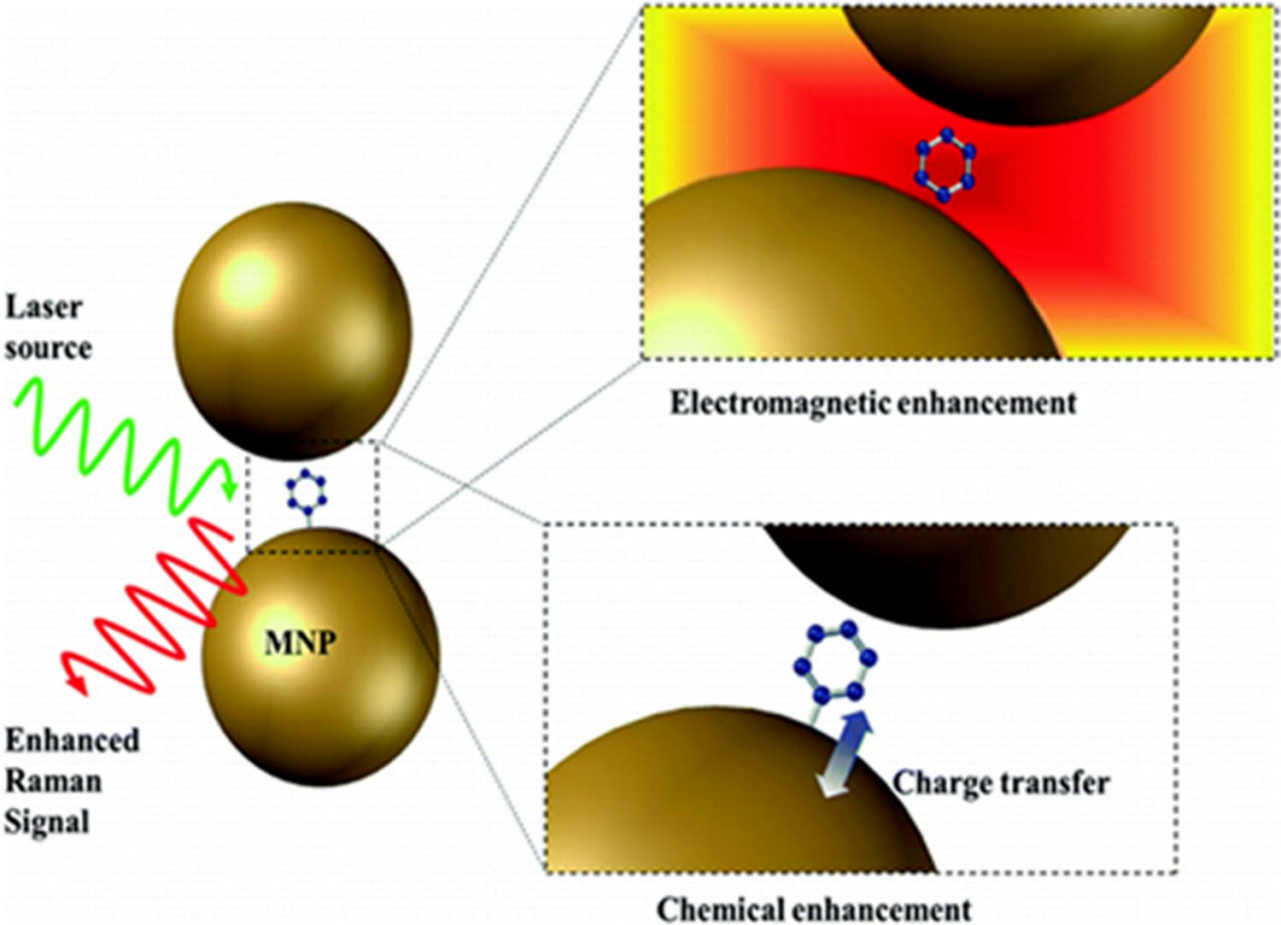
Illustration of the signal enhancement process in SERS (adapted from George (2020))

The use of conventional Raman spectroscopy for sensitive viral detection has been limited by weak signal strength (due to the low Raman scattering cross-section of biological samples). The low Raman scattering cross-section necessitates a longer signal acquisition time that often leads to sample damage (George 2020). This difficulty can be mitigated via the use of plasmonic nanostructures and consequent enhancement in the Raman signal, now popularly known as the surface-enhanced Raman spectroscopy (SERS) technique (Tadesse et al. 2020). Although resonance Raman spectroscopy can be applied for obtaining the fingerprint information of the molecule at very low concentration, the requirement for wavelength matching of excitation wavelength with electronic energy level of the molecule limits the technique. Moreover, most biomolecules exhibit absorption in the UV range and exposure to the radiation at this wavelength for long durations can damage clinical samples (Shipp et al. 2017). Despite the ongoing research on various biological systems using coherent anti-Stokes Raman scattering (CARS) technique, the increased complexity of the instrumentation precludes this technique as routine tool in clinical analysis. The application of tip-enhanced Raman spectroscopy (TERS) technique has been also restricted in biological sciences due to the complexity in fabricating reproducible tips (Huang et al. 2015). Raman tweezers have been associated with expensive and bulky instrumentation, which is unfavorable for point-of-care applications in clinical settings. Among the reported literature for SARS-CoV-2 detection using Raman-based techniques, the SERS technique has shown high promise for routine viral detection with satisfactory detection limit (George et al. 2018). The low Raman scattering cross-section, especially in the case of biological samples, can limit the applications of Raman spectroscopy for bioanalytical sensing purposes. To mitigate this, in SERS, the scattered light signal is improved by keeping analyte molecules in the proximity of the plasmonic metallic nanoparticles or nanostructures (George 2020; George et al. 2018; Barkur and Chidangil 2019; Xia et al. 2019; Hasna et al. 2016). The high sensitivity of the SERS technique is originated from the contribution of both electromagnetic and chemical enhancement mechanisms (Fig. 4). The concept of SERS, enhancement mechanisms, various fabrication methodologies employed for SERS substrate preparation and applications has been recently described (George 2020).

At present, a wide range of methodologies and protocols are being adopted to fabricate the SERS substrate, ranging from colloidal assisted approaches to advanced lithographic techniques (Shiohara et al. 2014). The plasmonic colloidal particle assisted SERS substrate fabrication technique relies on self-assembly or aggregation of the plasmonic particles, together with analyte molecules. The junction between the particle acts as hot spots to create large localized electromagnetic field to facilitate improved Raman scattering. Such approaches are reportedly able to detect analyte samples even at single molecule level (Shiohara et al. 2014). By varying the shape of the particles, the hot spot arrangement and consequently plasmonic field assisted Raman scattering signal enhancement can be varied to achieve improved signal strength. For example, anisotropic particles (e.g., star shaped or rod shaped) create higher field strength at the sharp edges of the corners and provide better signal strength for the molecules adhered there (Shiohara et al. 2014).

Gold nanorods are one of the most preferred structures since its optical properties can be highly tuned over a broad wavelength range based on its shape. Modifying the length to diameter (aspect ratio) of these nanoparticles can facilitate changes in surface plasmon band, which helps to utilize it for diverse applications. Another popular nanoparticle shape employed in SERS studies is the star-shaped particles (e.g., AuNS). The spikes of the NSs serve as an effective nanoantenna, which results in obtaining high electromagnetic field enhancement at the edge of each tip, leading to the creation of multiple hot spots within an individual nanoparticle. Owing to such improvements in Raman scattering of Ag nanoparticles, Ag nanostructures of different shapes (such as triangles, rods, and spheres) are employed in the SERS studies (Shiohara et al. 2014). Of these, the highest SERS enhancement is observed for star-shaped particles as compared to rod shaped and spherical Ag NPs due to localized electromagnetic field enhancement at hotspot between corners. Additionally, the spherical Ag NPs have the resonance band located at 400 nm whereas gold nanotriangles and nanostars have been found with multiple plasmon modes with a strong plane dipole band that allow resonance excitation of the Raman bands (Rycenga et al. 2011). Immobilization of the NPs on the substrate support material using linker molecules is a common practice (Toderas et al. 2007; Su et al. 2011). It is pertinent to note that Raman scattering is inversely proportional to the distance between the analyte and hot spot as I ~ 1/D^12^ (Ambartsumyan et al. 2020). Therefore, the proximity of the hotspot and analyte molecules are of paramount important in SERS studies. The SERS signal of the analyte reduces to zero at 3 nm from the hot spots (Dieringer et al. 2006; Masango et al. 2016). In the case of nanostructure-assisted SERS studies, the position of hotspots is fixed and thus the interface and molecule–substrate interaction have a pronounced effect on the SERS signal enhancement. The potential of anisotropic Ag nanoparticles (Ag NRs) has already been demonstrated to detect various viruses like adenovirus, human immunodeficiency virus, rhinovirus, and pathogens. Through integration of the improved stability of silver nanoparticles and enhancement factor of Ag nanoparticles, Au/Ag multilayered NRs arrays have been employed for the influenza A virus detection at very low concentrations (Ambartsumyan et al. 2020). The detection of viruses by the SERS technique can be made even more sensitive and specific by using reporter molecules (indirect detection method) attached with a sandwich immunoassay (Demirel et al. 2018). This route consists of a SERS tag, made up of a Raman reporter molecule and the recognition element, which is a specific antibody (detection antibody) confined on SERS-active nanoparticles substrate, and a template, called the capture substrate, which is functionalized with a linker antibody (capture antibody) to bind the antigen–SERS tag complex. Quantification is done by tracking the Raman signal from the reporter molecule before and after the SERS tag interacts with the capture element (Savinon-Flores et al. 2021) (Demirel et al. 2018).

Due to its flexibility, ease of use, and low cost, lateral flow immuno assay (LFIA) strips have been regarded as a suitable choice for POC diagnostics (Khlebtsov and Khlebtsov 2020). These sensors typically rely on visual readout avoiding any requirement for optical or electronic readout components. Efforts have been made to incorporate SERS tags with LFIA strips to improve the otherwise low sensitivity and limited quantitative/qualitative detection associated with conventional LFIA technique (Khlebtsov and Khlebtsov 2020). Kim et al. provided an in-depth review of Raman scattering-based lateral flow assay for infectious disease with reference to point of care applications. They have shown the efficacy of SERS multiplexed with lateral flow assay system in comparison with lateral flow immune assay (Kim et al. 2021b). Using SERS nanotags instead of solely gold nanoparticles can considerably enhance the Raman signals at hotspots, which can resolve the sensitivity issues in the standard LFIA detection. Use of Fe_3_O_4_/Ag magnetic SERS tags within the LFIA strips has facilitated an improved detection limit of 50 and 10 PFU/mL (plaque-forming units/mL) for influenza A H1N1 virus and human adenovirus (HAdV), respectively (Wang et al. 2019). In a similar work, multi-branched gold nanostars have been employed as SERS tags to provide a limit of detection (LOD) of 6.7 ng/mL for influenza A nucleoprotein, which shows a ~ 300-fold higher sensitivity as compared to conventional LFIA strips (Maneeprakorn et al. 2016). Hamad-Schifferli et al. developed a novel SERS-LFIA technique for the differentiation of the Zika virus and dengue virus nonstructural protein 1 (NS1) biomarkers (Sánchez-Purrà et al. 2017). That sensor employed SERS-encoded Au nanostars which resulted in the improvement of LOD ~ 7 and ~ 15 fold for dengue and Zika virus, respectively. Jeon et al. have developed a SERS-LFIA immunoassay, which can resolve the thermal stability concerns in the normal LFIA strips for their application in tropical regions. Using silica-encapsulated gold nanoparticles as tags for viral detection, the sensing strips were stable and reproducible at ~ 45 °C, whereas the solely gold nanoparticle-based LFIA strips suffered sensitivity issues (Jeon et al. 2020). Tang et al. reported a novel core–shell structure material, AuAg4 − ATP/AgNPs, as a Raman probe for the detection of avian influenza A (H7N9) virus with an estimated LOD of 0.0018 HAU (hemagglutination units) (Xiao et al. 2019). Similarly, Au-core Ag-shell nanoparticles were used for the detection of rotavirus in fecal samples using a SERS immunochromatographic assay (ICA) (Zhang et al. 2021c) with this assay able to quantitatively detect the virus in the range of 8–40,000 pg/mL with an LOD obtained as 8 pg/mL. Specific viral targets can be recognized by oligonucleotide aptamers and because of this the technique is gathering more interest recently (Kim et al. 2010). The popularity of the oligonucleotide-based SERS technique can be attributed to the ease of synthesis and purification process in addition to the potential for tagging and easy conjugation with metal and carbon-based nanostructures (Kim et al. 2010; Muhammad & Huang 2021). Moreover, the chaotic orientation, signal interference with SERS signal, and low thermal stability of antibodies compared with aptamers are promoting aptamer-based SERS platforms for virus detection (Tadesse et al. 2020; Tort et al. 2012).

## Comparative studies using the SERS technique for investigation of different viruses

**Fig. 5:**
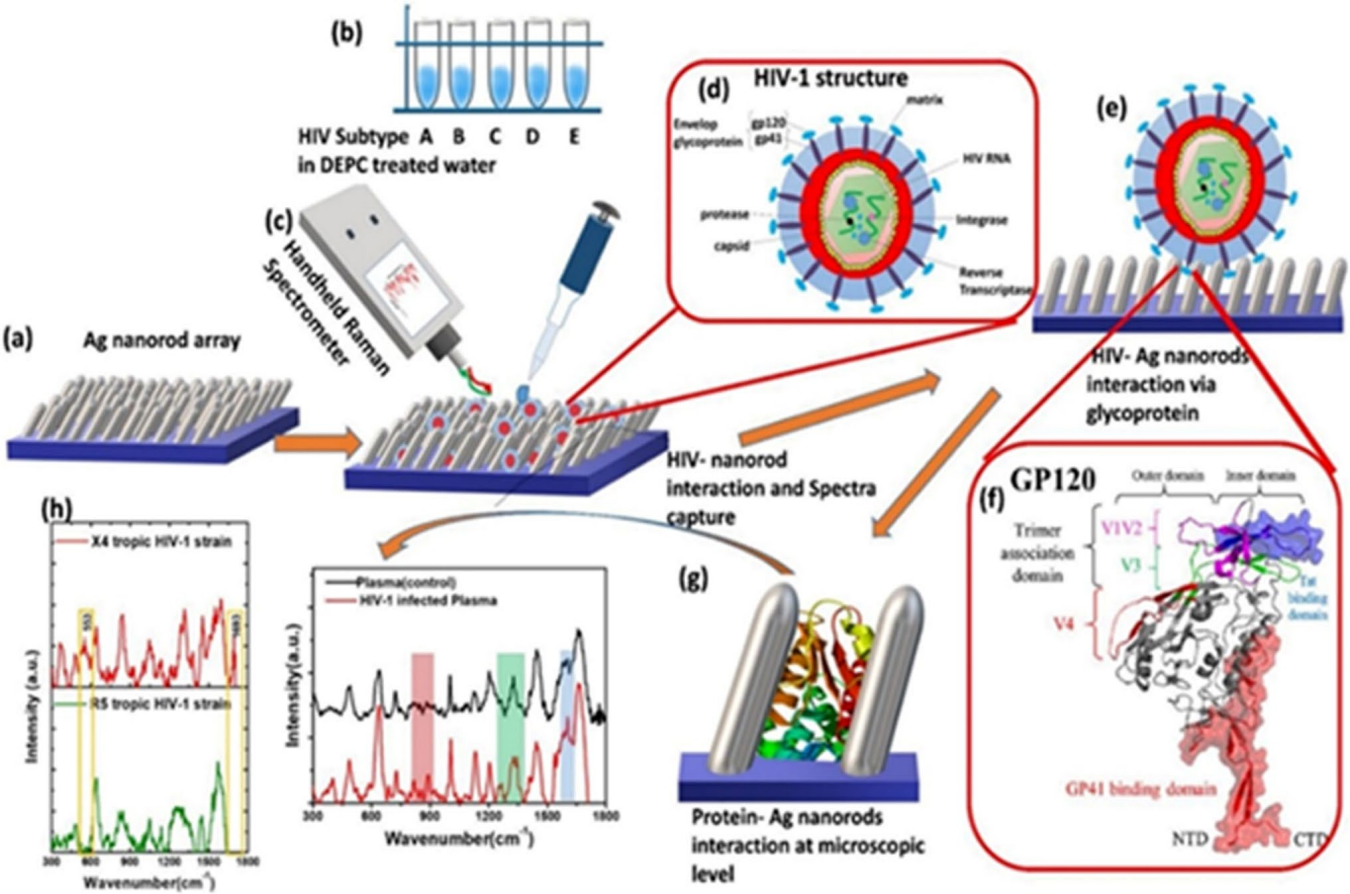
Fabrication and working principle ((**a**)–(**h**)) of rapid handheld SERS platform for HIV detection (adapted from Yadav et al. (2021))

**Fig. 6:**
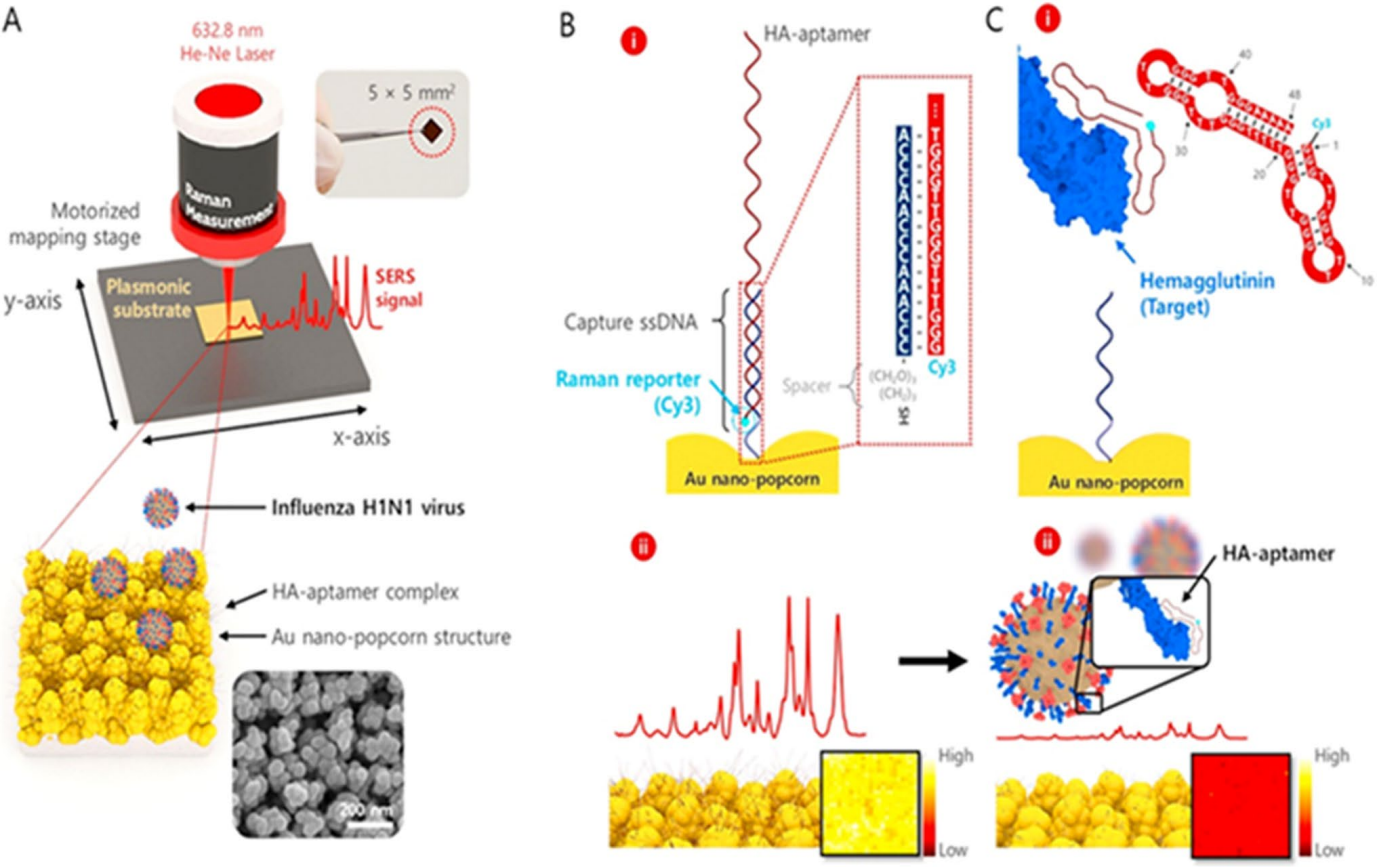
Schematic diagram demonstrating SERS detection of influenza virus (adapted from Chen et al. (2020))

Recently, Amin Hadi et al. reviewed the applications of SERS in detection of biomaterials with special emphasis on COVID-19 detection. The article covers a vast spectra of details including the basic working principal of SERS to the proof of concept study demonstrating the applications in virus diagnostics (Eskandari et al. 2022). Yadav et al. proposed the use of a silver nanorod array, fabricated via the glancing angle deposition technique, for detection of the human immunodeficiency virus (HIV). Using a handheld SERS platform, this method was capable of selectively detecting HIV-1 subtypes in virus spiked blood plasma with minimal sample volume requirement (5 µL) (Yadav et al. 2021). A schematic diagram of the viral detection protocol using this SERS-based biosensor is shown as Fig. 5. SERS nanoprobes with gold shell-isolated nanoparticles were fabricated and conjugated with Nile blue dye used as the Raman reporter to achieve detection of Zika virus antigen at a concentration as low as 10 ng/mL. Further to this, the overlap of the Nile blue absorption band with the Raman probe laser excitation at ~ 633 nm can produce surface-enhanced resonance Raman scattering (SERRS), to enable accurate antigen detection (Camacho et al. 2018). Ryes et al. have developed a protein aggregation SERS detection scheme for detection of enterovirus from protein-rich samples (Reyes et al. 2017). In that study, Raman peak intensities at 510, 670, and 910 cm^−1^, which are dependent on the aggregation state of a specific gold nanoparticle-based bioconjugate, were analyzed to detect the presence of enterovirus 71. Others have demonstrated non-lithographically prepared metal/polymer nanostructured SERS substrates, which can carry out the detection of respiratory syncytial virus and coxsackievirus (Demirel et al. 2018). Antisense oligonucleotides (ASO) having complementary sequences with unique sites on viral genomes were considered as promising recognition elements in the Raman spectroscopic studies of viruses (Ambartsumyan et al. 2020) (Fig. 6).

The synthesis of ASO is relatively straightforward, and it includes the sequencing of the target virus genomes and the selection of a unique sequence for a specific viral strain of interest (Schubert and Kurreck 2006; Lenartowicz et al. 2016). Negri et al. have used an array of DNA oligonucleotides functionalized on a silver-based nanorod substrate for detecting generic markers linked with high pathogenicity in the influenza virus (Negri and Dluhy 2013). Another example demonstrating the potential of viral detection by SERS aptamer probes using the recently reported SERS imaging-based assays is illustrated in Fig. 6 (Chen et al. 2020). In this case, Chen et al. fabricated a 3D nano-popcorn substrate and functionalized it with thiolated captured DNAs for hybridizing Cy3-labeled aptamer probes for detecting influenza virus. The coupling between the virus and the aptamer probes resulted in the reduction of Raman signal intensity (Fig. 6C), which has been exploited for the quantitative detection of the virus. The possibility of influenza virus detection was also proposed by measuring the SERS signal from the aptamer-nucleoprotein complex generated after the binding of the anti-influenza aptamer to viral nucleoprotein (Negri et al. 2012).

**Fig. 7:**
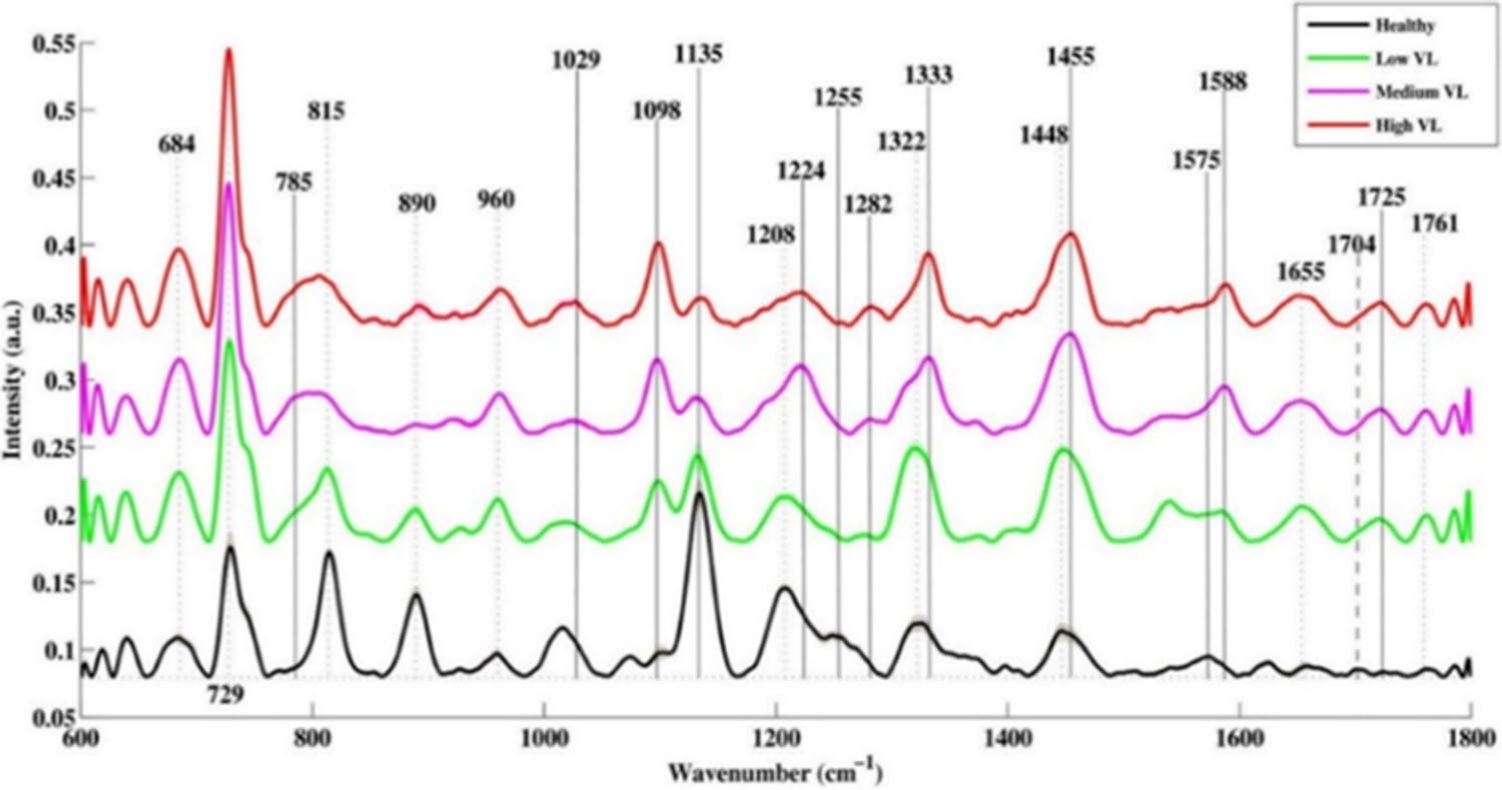
SERS spectra of healthy and hepatitis C infected serum samples with different viral load (VL) (adapted from Kashif et al. (2020))

The SERS spectral signatures of the aptamer-nucleoprotein complex were able to effectively discriminate the selective binding of the aptamer-nucleoprotein from that of the aptamer alone. The use of gold-coated paramagnetic nanoparticles was reported as effective SERS substrates for targeting the DNAs derived from virus genomes (Zhang et al. 2012). Liu et al. have also employed graphene oxide-gold nanorods composite for the detection of hepatitis B surface antigen in serum with a limit of detection ~ $0.05\;\mathrm{pg}\cdot\mathrm{mL}^{-1}$ (Liu et al. 2018). Lee et al. have reported a study demonstrating the use of SERS substrates for the detection of the HIV-1 within 5 s. Highly ordered Au nanodots deposited electrochemically on to indium tin oxide (ITO) substrates were able to detect HIV down to 35 fg/mL (Lee et al. 2015). In 2006, the literature reported a study using silver nanorod array substrates which was capable of rapid, differential detection of respiratory viruses and viral strains (Shanmukh et al. 2006). Two years later, the same group has reported SERS combined with chemometric tools for the reliable discrimination of closely related respiratory syncytial virus (RSV) strains and extended the work for human rotavirus detection and classification based on their genotypes (Shanmukh et al. 2008; Driskell et al. 2010). Lim et al. have also used SERS for influenza virus detection by exploiting enhanced spectral markers of their unique surface proteins/lipids generated with the aid of gold nanoparticle substrates. The technique was capable of differentiating non-influenza viruses from the influenza virus, and in addition, discrimination among different sub-types of influenza viruses. Determination of the presence of viral RNA mutations is important from both the preventative and therapeutic perspectives (Lim et al. 2015). The potential of the SERS technique to probe RNA mutations in the influenza A virus genome was shown using thiolated DNA hairpin functionalized gold nanostars (Dardir et al. 2020). In this case, a Raman active fluorescent dye was attached to 3’ terminus of a DNA sequence. The DNA hairpin structure was extended or folded in the presence or absence of viral RNA targets, respectively, causing the fluorophore to be close to the nanoparticle surface creating a kind of “OFF–ON” switching of the Raman signals. The detection was found to be sequence-specific, and the target detection at a single particle level was demonstrated. In addition, the signal recovery was found to be in correlation with the number of genetic mutations. Conventional cell lysing, RNA extraction, and preamplification procedures can be avoided in this SERS-based approach, which altogether yield a considerable reduction in detection cost and time (Dardir et al. 2020).

**Fig. 8:**
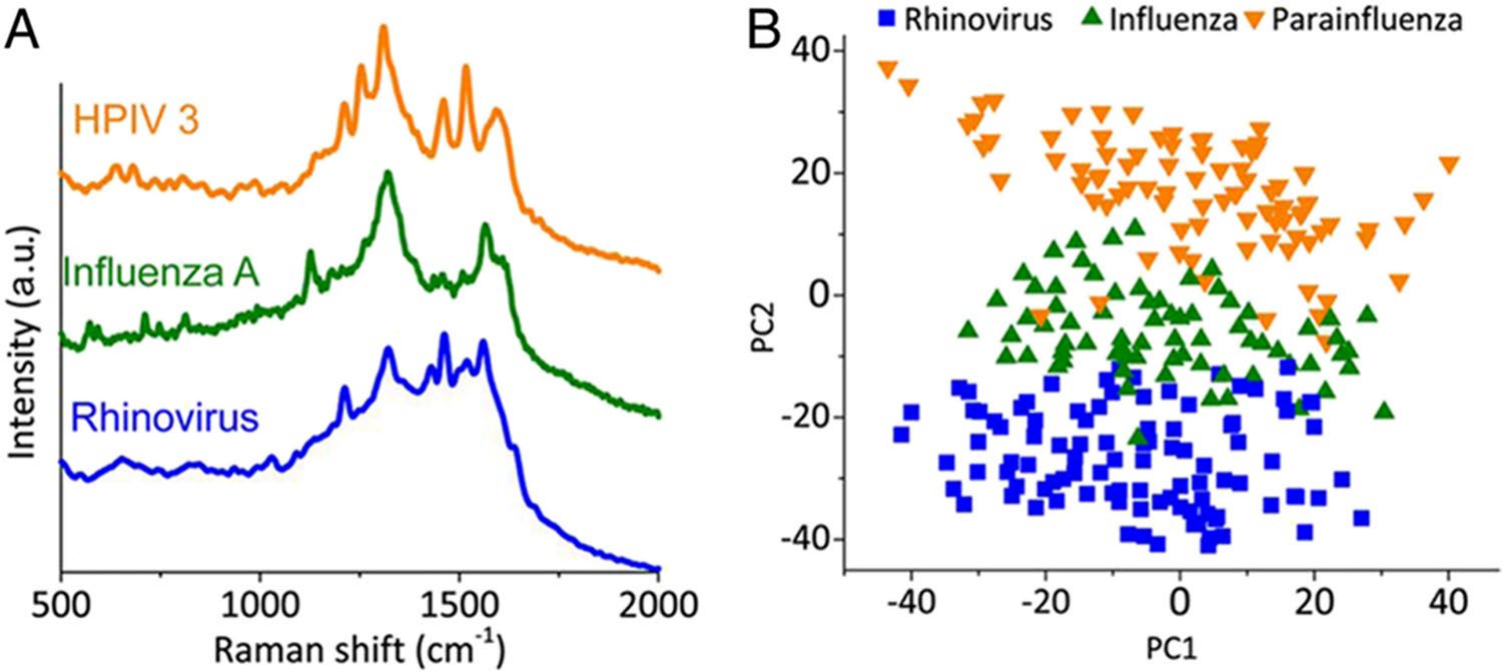
**A** Raman spectra of respiratory virus. **B** PCA plot of Raman spectral data (adapted from Yeh et al. (2020))

Gahlaut et al. have used a handheld Raman instrument, which enabled the detection of nonstructural protein (NS1) features from serum samples. Detection of NS1 protein, which is a secretory component of the virus, was utilized for the dengue diagnosis from serum (Gahlaut et al. 2020). Another recent work has demonstrated the possibility of hepatitis B viral load estimation and quantification from PCR products. Gel documentation systems (GDS) usually employed for the analysis of PCR products involve costly instrumentation, reagents, and lengthy UV exposure which can adversely impact the fluorescence of the bands. Batool et al. have analyzed the PCR product of viral RNA extracted from the hepatitis B patients using the SERS technique and the use of multivariate analytical tools such as principle component analysis (PCA), partial least squares-discriminant analysis (PLS-DA), and partial least squares regression (PLSR) resulted in a measured sensitivity of 89% and specificity of 98% (Batool et al. 2021). SERS measurements performed using silver nanoparticle solution on serum samples collected from hepatitis B virus-infected patients have shown an enhancement in the L-arginine Raman line at 493 cm^−1^ and a reduction in the nucleic acid bases in comparison with healthy volunteers (Lu et al. 2018). In addition, the peak assigned to saccharide at 889 cm^−1^ was found to be increased whereas the band of valine at 960 cm^−1^ decreased in diseased patients. This has been attributed to the saccharide metabolism disorder and the inhibition of the process of glycolysis (Lu et al. 2018). Additionally, markers for nonstructural protein (NS1) detection were identified from human saliva, which can be useful for monitoring viral infections (Othman et al. 2017). Recent studies have reported that SERS is capable for the detection of hepatitis C as well as quantification of viral load (VL) from blood serum samples (Nasir et al. 2021; Kashif et al. 2020). The peak present at 785 cm^−1^ originating due to the uracil base has been identified as a major marker for viral RNA, which is absent in normal serum samples, as evident from the spectra shown in Fig. 7. The increase in viral load has also resulted in the decrease in lipid contents seen in bands attributed to the lipid stretching at 1135 cm^−1^ and 1255 cm^−1^ (Fig. 8). The increase in RNA/DNA content in the clinical sample is in accordance with viral load measured from the Raman band at 1098 cm^−1^.

Microfluidic platforms are associated with benefits that include low sample volume, programmable sample handling, and automatic assay procedures (Bai et al. 2020). Incorporating the SERS technique into microfluidic platforms can pave way for the generation of automatic high-throughput on-chip detection (Bai et al. 2020; He et al. 2020; Ma et al. 2021; Burtsev et al. 2021; Gao et al. 2019). Recently, a microfluidic Raman platform that enables viral detection from clinical samples within a few minutes has been developed. The handheld device named “Virrion” allows for the capture of viruses depending on their size by use of a carbon nanotube forest arrays doped with gold nanoparticles patterned in a microfluidic channel, with later viral recognition from the patient sample executed using SERS followed by machine learning algorithms (Yeh et al. 2020). Raman spectra of respiratory viruses recorded using Virrion are shown in Fig. 8A and the PCA results (Fig. 8B) of the data were found to be effective in differentiating the viral strains. Hepatitis B virus antigen in blood plasma down to a level of 0.01 IU/mL was detected in a microfluidic system using SERS immunoassay (Kamińska et al. 2015). In that study, Kaminska et al. utilized a fuchsin-labeled immune-gold nanoflower together with SERS substrate for carrying out quantitative investigation on the virus with high sensitivity and selectivity. An Au–Ag coated GaN substrate embedded in the polycarbonate microfluidic device served as a SERS active substrate in this work (Kamińska et al. 2015). In a related study, Wang et al. have integrated digital microfluidics (DM) technology with the SERS technique for carrying out the automated and quantitative detection of avian influenza virus H5N1 in human serum (Wang et al. 2018). In that work, microfluidic chip was connected to a driving device, which can control the transport of a sample droplet to and from the original reservoirs upon application of an electric field to an array of electrodes on the substrate. The DM-SERS technique has shown a detection limit of 74 pg/mL with a shorter assay time (< 1 h) and low reagent consumption (~ 30 µL) with respect to conventional ELISA technique. A recent study has shown the ability of SERS technique to detect drug-resistant influenza virus in human nasopharyngeal aspirate using antibody mediated assays (Kim et al. 2021a).

Characterization of different viruses can be done via SERS by using specific reporter molecules attached to the immunoassay (Sivashanmugan et al. 2013). Viruses bind to a specific antibody functionalized on the nanostructures and hot spot formation happens. Characterization and quantification are done by tracking SERS signals from the reporter molecule before and after the SERS reporter molecule bind with the target analytes. Therefore, finding a particular Raman band or peak for each type of virus is not straightforward. In one study, Au/Ag multilayered nanorod arrays were prepared through the focused ion beam technique for the detection of the influenza A virus strain. The most intense peak for H2N2 was observed at 725 cm^−1^ (adenine), whereas the bands of adenine and tyrosine at 854 and 730 cm^−1^ were found to be prominent in the case of H3N2 (Sivashanmugan et al. 2013). Similarly, the intensity of the signal located at 1643 cm^−1^ in the case of A (H1N1) was more prominent. It has been observed that there are similarities in signal response between the SARS-CoV-2 virus and SARS-CoV-2 spike protein (YeonáYi & HyunáChung 2016). In a SERS study of different strains of the rotavirus, each virus strain generated similar SERS spectra with the major bands appearing at 1003, 1030, 1045, and 1592 cm^−1^. However, the SERS spectral fingerprints could differentiate samples as rotavirus-negative or rotavirus-positive. Using partial least squares regression (PLSR) analysis, SERS spectra for eight different rotavirus strains were able to classify each according to G and P genotype with an accuracy of more than 96% (Driskell et al. 2010). The details about the SERS substrates, the virus which was studied, and their corresponding Raman bands were given in Table 1.

**Table 1:** Different SERS substrates for the study of various viruses with assigned Raman bands

SERS substrate/Raman	Virus name/strain detected	Assigned peak (cm^−1^)	Corresponding vibrational made	Reference
Klarite (Au on Si)	Norovirus MNV4	844	Tyrosine	Fan et al. (2010)
		921, 937, and 943	C–COO^−^ stretch	
		1001	Phenylalanine (symmetrical ring breathing)	
	Adenovirus MAD	1008, 1022	Phenylalanine (in-plane C–H bending)	
		1047	C–N stretch	
		1129	C–N and C–C stretch	
	A variety of enveloped viruses	540	S–S stretch	
		640	Tyrosine (skeletal)	
		720, 744	Adenine	
Au/Cu hollow nanocones of microbowls (HNCMB)	Adenovirus type 5	1015	Carbohydrates for solids	Zhang et al. (2019)
	Coxsackievirus type 3	~ 1041–1062	C–N stretching	
Ag nanorod	Adenovirus	~ 643–656	Guanine	Shanmukh et al. (2006)
		~ 719–730	Adenine	
	HIV	~ 1448–1454	CH_2_ deformation	
		~ 1523–1597	ν_a_ COO^−^ Trp	
	Rhinovirus	~ 848	Tyrosine	
		1002	Phenylalanine	
Gold “virus traps” nanostructure	SARS-CoV-2 spike protein	568	Amide V	Yang et al. (2021)
		884	Tryptophan	
		1296	Amide III (α helix)	
	SARS-CoV-2	645	Tyrosine	
		884	Tryptophan	
		950	*v*(C–C, α-helix)	
		1027	Phenylalanine	
		1215	Trp and Phe, *v*(C-C_6_H_5_)	
		1445	Amino acid, *v*(-CH_2_)	
Au/Ag multilayered nanorod arrays onto silicon crystal	Influenza A(H1N1) Spanish flu	1643		Sivashanmugan et al. (2013)
	Influenza A(H2N2)	725	Adenine	
	Influenza A(H3N2)	854 and 730	Tyrosine and adenine ring	
SERS-gold nanoparticles (GNPs)	A/WSN/33 H1N1 + hemagglutinin (HA) and neuraminidase (NA)	737, 1331, 1467, and 1625		Lim et al. (2019)
	A/California/04/2009 H1N1 + hemagglutinin (HA) and neuraminidase (NA)	609, 720, and 1625		
SERS (Ag nanorods array)	Dengue	1005, 1395	Benzene ring breathing (phenylalanine), stretching of the COO-bond	Gahlaut et al. (2020)
SERS (Ag colloidal solution)	Hepatitis B	733, 957, 1032, 1419, 1566	Adenine, deoxyribose, guanine, guanine, guanine	Batool et al. (2021)
SERS (Ag colloidal solution)	Hepatitis B	493, 889	L-arginine, saccharide	Lu et al. (2018)
SERS Ag nanoparticle solution	Hepatitis C	704, 811, 1036	Ring deformation of cytosine, C–O–P–O–C bands in RN, stretching of C–O and C–C	Nasir et al. (2021)
SERS	Hepatitis C	785, 1029	Ring breathing modes in the RNA base/uracil, O–CH_3_ stretching of methoxy group	Kashif et al. (2020)

## SARS-CoV-2 detection

Many research groups have exploited Raman spectroscopy-based modalities for the detection of SARS-CoV-2 (Yang et al. 2021; Pramanik et al. 2021). Owing to its low scattering cross-section, detection of the SARS-CoV-2 virus is preferentially carried out using the SERS technique as compared to the traditional Raman spectroscopic technique. A SERS biosensor for detection of viruses was demonstrated using an ACE2-functionalized structured gold nanoneedle array (GNA) as “virus traps” (Yang et al. 2021). Using this technique, four proteins, namely, SARS-CoV-2 S protein, SARS-CoV-2 nucleocapsid protein, SARS-CoV S protein, and human ACE2 protein, could be characterized by measuring corresponding SERS spectra. The SERS spectra of those four proteins exhibited three characteristic peaks of SARS-CoV-2 S at 568 (amide V), 884 (trp), and 1296 cm^−1^ (amide III α helix) were apparent in all the recorded spectra. However, statistical analysis of the recorded Raman spectra using the PCA technique showed the potential of the technique to differentiate between individual spike proteins, namely, SARS-CoV-2 and SARS-CoV. Strains of the SARS-CoV-2 virus were analyzed in terms of their spike protein and nucleocapsid proteins, commonly represented as V_S_ and VN, respectively (Yang et al. 2021). It was reported that the spike protein features exhibited at 568, 644, 884, 950, 1027, 1310, and 1445 cm^−1^ of the SERS spectra of SARS-COV-2 virus suggesting that the spike proteins are the major contributors to the Raman signature of the virus (Yang et al. 2021). Comparative studies of the inactivated and live SARS-CoV-2 virus exhibited substantial differences in spectral features, especially at 645, 884, 950, 1027, 1215, and 1445 cm^−1^ largely associated with the spike proteins. SERS signals of spike protein collected from a region of an area of 20 × 14 μm^2^ exhibited a relative standard deviation (RSD) for the Raman bands intensity (569 cm^−1^ and 1027 cm^−1^) of only 10.7% and 4.9%, indicating a high reproducibility. Using this technique binding between the ACE2 and SARS-CoV-2S is reported to be much more efficient than that between ACE2 and SARS-CoV-S. In addition, the study also elucidated that the viral infection process for SARS-CoV-2 is faster in comparison with the SARS-CoV version (Yang et al. 2021).

In a study based on colorimetric change, Au nanoparticles were functionalized with anti-spike antibody and used for rapid identification of SARS-CoV-2 antigen/virus within 5 min (Pramanik et al. 2021). Exhibiting rapidity, high selectivity, and high sensitivity, the SERS technique was used with 4-aminothiophenol as a reporter molecule adhered to the GNPs via an Au–S bond. When GNPs were added to the antigen or virus particles, antigen–antibody binding occurs which leads to the GNP aggregation and consequent color variation from pink to blue (Pramanik et al. 2021). Color change was observed by eye over a range bounded by a minimum concentration of 1 ng of COVID-19 antigen and 1000 virus particles per mL. SERS-based pseudo-SARS-CoV-2 virus detection using the anti-spike antibody and 4-aminothiophenol linked GNPs relies on the fact that in the presence of virus, the nanoparticles accumulate on the virus surface due to spike protein-antibody interaction. As the size of the virus is in the order of 120–150 nm, many functionalized gold nanoparticles can aggregate over each virus particle and form several hotspots and thus contribute to improved SERS signal. This interaction process took a maximum of 5 min to achieve the desired result and the Raman peak at 1078 cm^−1^ of anti-spike antibody and 4-aminothiophenol-functionalized gold nanoparticles was taken as reference. The intensity of the SERS peak at 1078 cm^−1^ varied linearly with the concentration of virus particles of pseudo-SARS-CoV-2. A LOD of 18 virus particles of SARS-CoV-2/mL could be detected, which is better than the present gold standard reverse transcription–polymerase chain reaction (RT-PCR) test where the LOD is 40 virus particle/mL (Pramanik et al. 2021).

A Raman spectroscopy investigation of SARS-CoV-2 in 17 water samples using a portable Raman spectrometer demonstrated the on-site measurement capability of the technique, in addition to the performance evaluation of the disinfection as well as viral survival in an environmental sample (Zhang et al. 2021a). That study employed the ACE2 receptor functionalized on silver nanorod array for the detection of viral presence in water (Liu et al. 2021). The binding of receptor-binding domain (RBD) of SARS-CoV-2 spike protein on the sensor array resulted in the quenching of most of the spectral features of the ACE2 receptor in addition to a shift in the band from 1189 to 1182 cm^−1^. The study has been suggested as a potential alternative to monitor the viral presence in wastewater treatment plants and pipe networks (Zhang et al. 2021a).

**Fig. 9:**
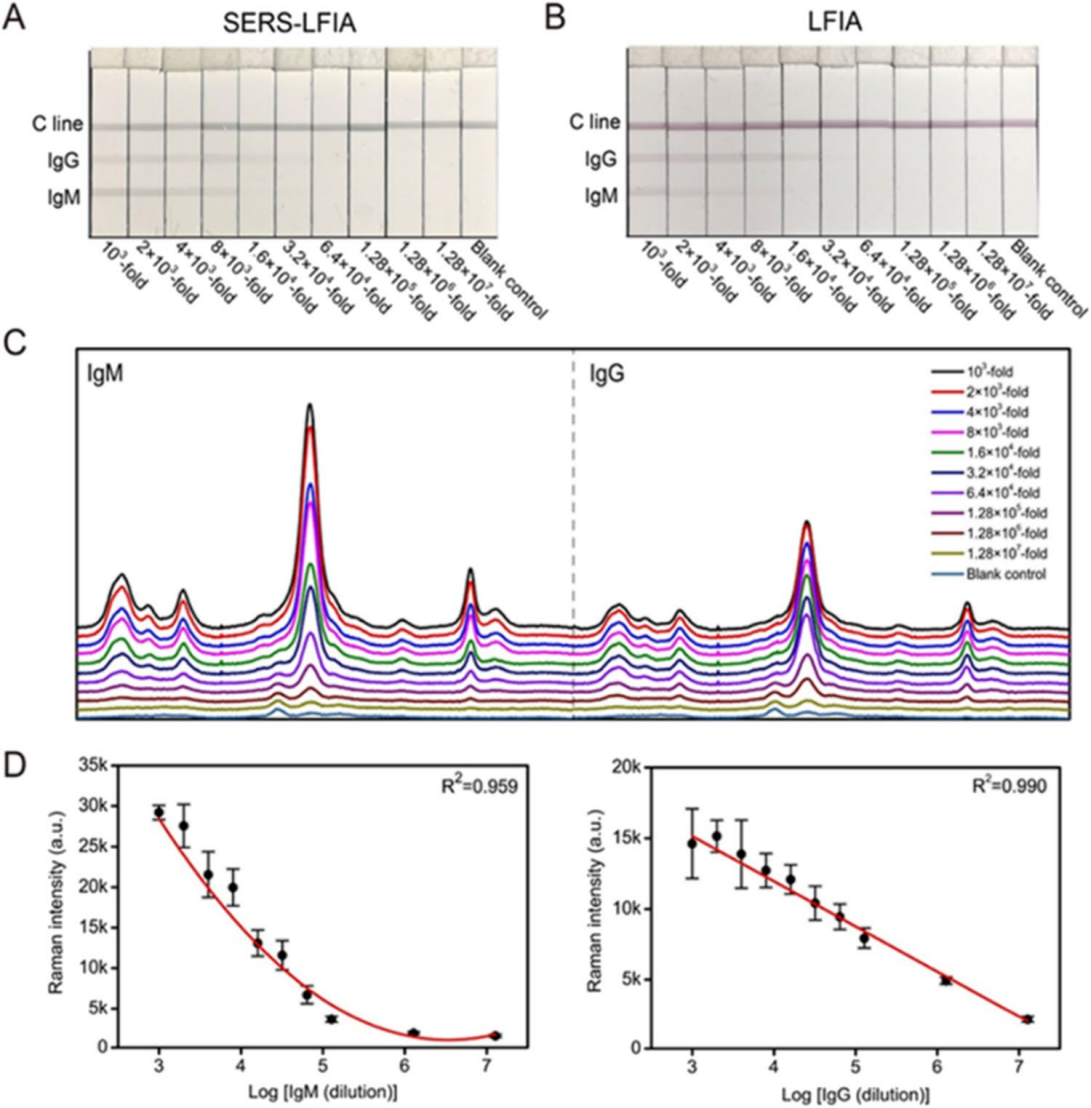
IgM/IgG detection using **A** SERS-LFIA strips and **B** Au NP-based LFIA strips. **C** Raman spectra obtained for IgM and IgG lines. **D** Raman calibration plot obtained for IgM and IgG considering the band at 1328 cm.^−1^ (adapted from Liu et al. (2021))

Even though LFIA methods have been reported for SARS-CoV-2 detection, the colorimetric analysis suffers shortcomings in quantitation and sensitivity (as discussed earlier). To resolve these concerns, Liu et al. demonstrated a fast and highly sensitive SERS-LFIA sensor for identifying SARS-CoV-2 antibodies in human serum with the experimental observations are summarized in Fig. 9 (Liu et al. 2021). They developed novel SERS tags by coating a complete Ag shell over an SiO_2_ core (SiO2/Ag) and conjugated this with viral spike protein for sensing purpose. The biosensor has been effective in the simultaneous detection of anti-SARS-CoV-2 IgG and IgM with excellent sensitivity as shown in Fig. 9 (as much as 800 times more sensitive than the conventional gold nanoparticle-based LFIA). The sensor performance was verified on 68 clinical samples with a demonstrated 100% accuracy and specificity of the SERS-LFIA. This technique can be advantageous for the screening of early infections, where the presence of antibodies in serum will be considerably lower.

**Fig. 10:**
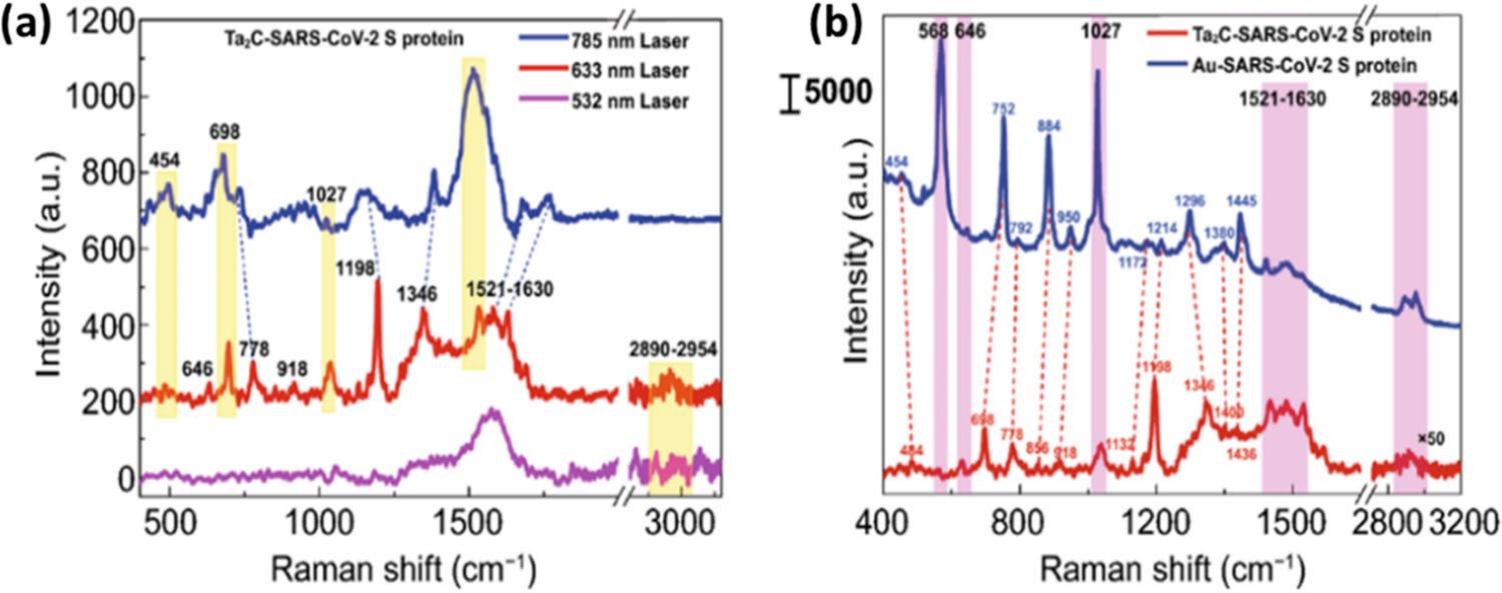
**a** Raman spectra of spike protein recorded with the different excitation wavelengths. **b** S-protein Raman spectra recorded using Ta_2_C NSs and Au NPS substrates (adapted from Peng et al. (2021))

Semiconductor-based SERS substrates have been utilized for ultra-sensitive detection of SARS-COV-2 protein (Peng et al. 2021). In this study, Ta_2_C MXene substrates have provided excellent SERS performance through photo-induced charge transfer (PICT) resonance enhancement and electromagnetic enhancement. This study demonstrated detection of SARS-CoV-2 spike protein, with a detection limit achieved as low as 5 × 10^−9^ M (Fig. 10). Somewhat similarly, Zhang et al. demonstrated a triple mode biosensing (colorimetric/SERS/fluorescence) approach for detection of SARS-CoV-2 RNA at femtomolar level within 40 min. The integration of multimodal approaches in biosensing can possibly increase the detection sensitivity and accuracy with reduction in false positive cases (Gao et al. 2021).

**Fig. 11:**
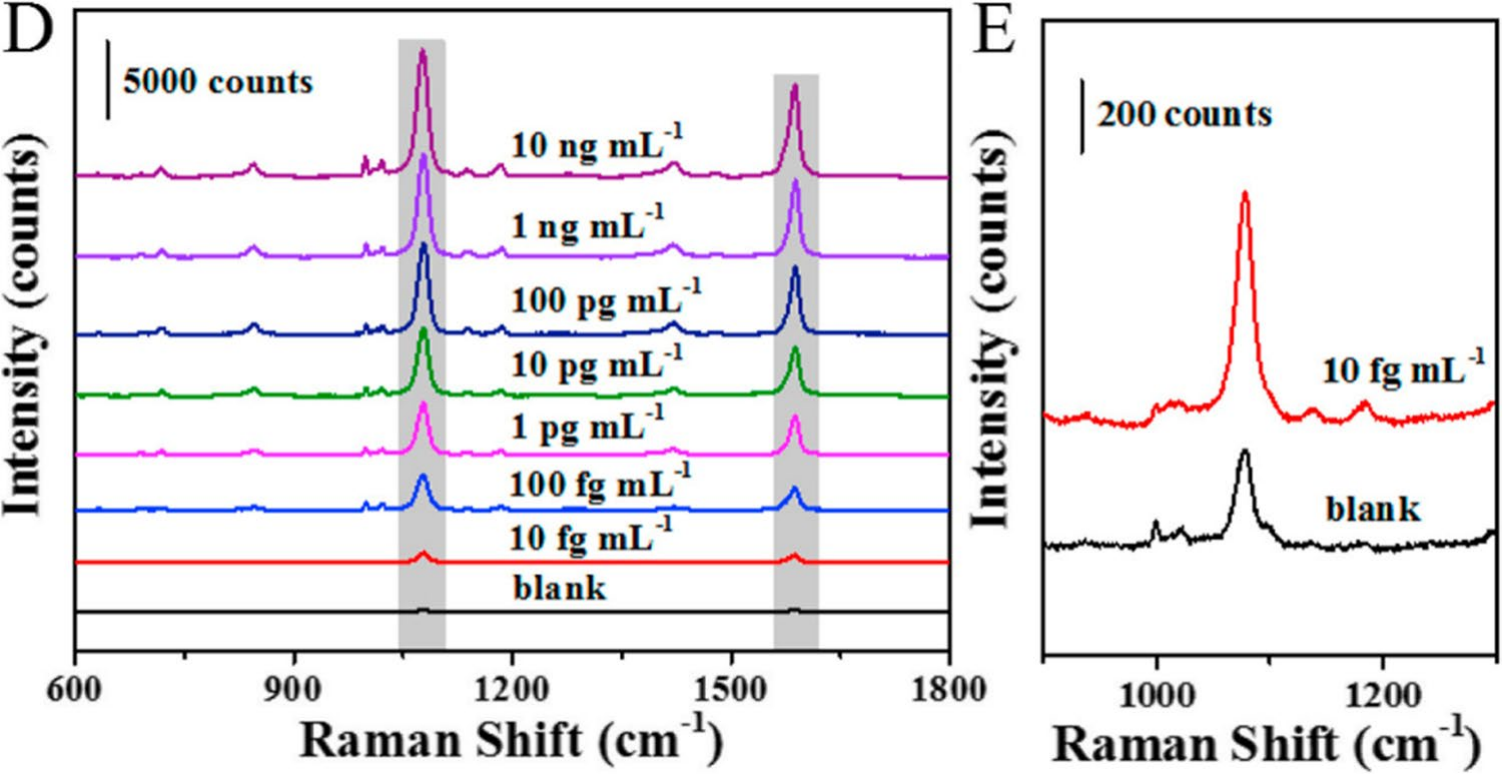
**D** SERS spectra obtained for various SARS-CoV-2 spike protein concentrations in saliva. **E** Comparison of Raman spectrum of spike protein in saliva and the blank signal (adapted from Zhang et al. (2021b))

Zhang et al. have demonstrated the possibility of SARS-CoV-2 spike protein detection from unprocessed saliva using SERS biosensing (Zhang et al. 2021b). SARS-CoV-2 spike antibodies conjugated to the SERS substrate fabricated via unique oil/water/oil three-phase liquid–liquid interfaces self-assembly process enabled ultrasensitive detection of SARS-CoV-2 spike protein n phosphate-buffered saline (0.77 fg mL^−1^) and untreated saliva (6.07 fg mL^−1^). The spectra obtained for different antigen concentrations in saliva have been shown in Fig. 11.

Stanborough et al. have fabricated an aptamer-based SERS sensor for SARS-CoV-2 antigen protein detection at subpicomolar concentrations (Stanborough et al. 2021). The silver nanoparticle surface-functionalized with thiolated aptamer has shown depletion of free N–H stretching band upon the formation of strong amide hydrogen bonds during the selective, specific binding to spike protein. Another study involving aptamer receptors has been reported by Chen et al. where DNA aptamers immobilized on gold nano-popcorn surface were employed for sensing spike protein within 15 min (Chen et al. 2021). Detection limit ~ 10 PFU/mL was obtained for this study performed using SARS-CoV-2 lysate spiked in clinical samples, a comparatively better sensitivity with respect to commercial immunoassay rapid kit (~ 300 PFU/mL). Quantitative detection of SARS-CoV-2 virus along with high selectivity from other respiratory viruses via SERS was also demonstrated using aptamer-based silver colloidal solutions (Zavyalova et al. 2021). Huang et al. explored the diagnosis of SARS-CoV-2 antigen from clinical samples within 20 min using the deep learning-based SERS approach (Huang et al. 2021). This analytical approach utilized the hot spots generated by an aggregated spherical gold nanoparticle array for targeting the S protein of SARS-CoV-2. Upon the combination of a residual neural network (RNN)-based deep learning model, the study performed using a portable Raman spectrometer has shown promising results from throat swabs with sensitivity, specificity, and accuracy of 83.3%, 92.5%, and 87.7%, respectively, highlighting its potential as an onsite, rapid screening tool. The possibility of integrating the whole device inside a vehicle and thus the development of a mobile platform for field-based viral detection using this SERS approach was proposed in this work*.*

**Fig. 12:**
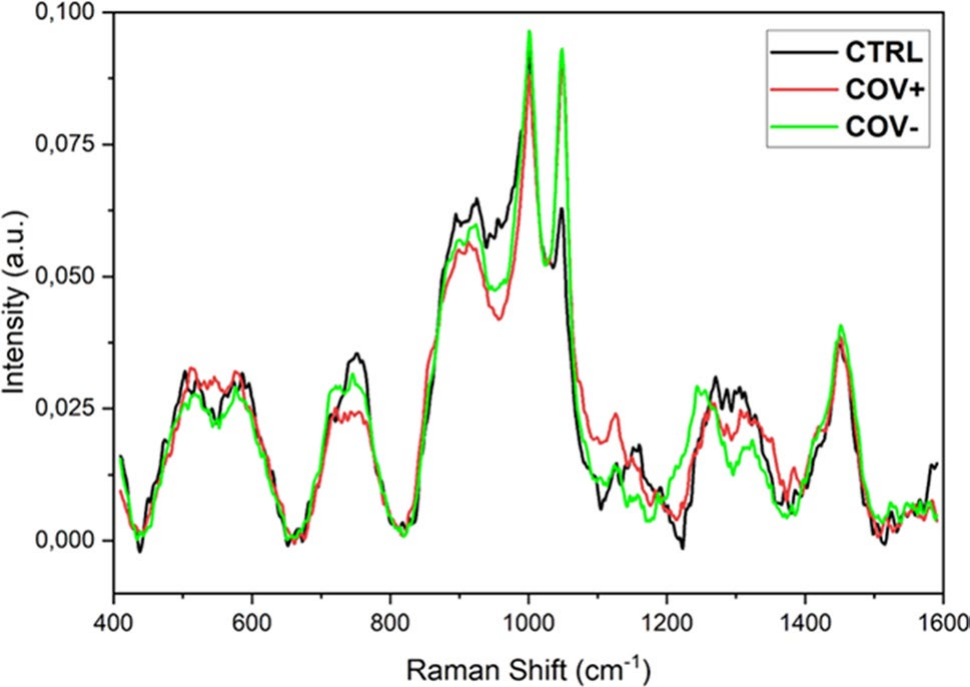
Raman spectra of healthy subjects (CTRL), subjects with viral infection (COV +), and subjects with history of viral infection (COV −) (adapted from Carlomagno et al. (2021))

Goulart et al. have employed the Raman technique for the diagnosis of COVID-19 by identifying the spectral features of immunoglobulins IgM and IgG present in human serum (Goulart et al. 2021). Raman measurements have shown an increase in the characteristic markers attributed to nucleic acids, tryptophan, and immunoglobulins for COVID-19 cases. The study carried out on 54 control and 40 COVID-19 clinical samples was able to discriminate positive cases from control with a sensitivity and specificity of 84.0% and 95.0%, respectively, upon PLS-DA analysis. Carlomagno et al. have conducted SERS investigations on saliva collected from SARS-CoV-2 infected patients (COV +), healthy persons (CTRL), and patients with COVID-19 history (COV −) (Carlomagno et al. 2021). The spectral patterns obtained from three different classes are given in Fig. 12. The peaks present at 1048 and 1126 cm^−1^ have been found higher in patients infected with the virus and also in patients infected in the past with respect to control samples. The enhancement in these Raman peaks is indicative of aromatic amino acids (tryptophan and phenylalanine) abundant environment, which have been identified in the literature as one of the characteristic features of viruses of the coronavirus family. The spectral features obtained from saliva were sufficient enough for the discrimination of COVID-19 patients with accuracy, sensitivity, and specificity above 95%.

**Fig. 13:**
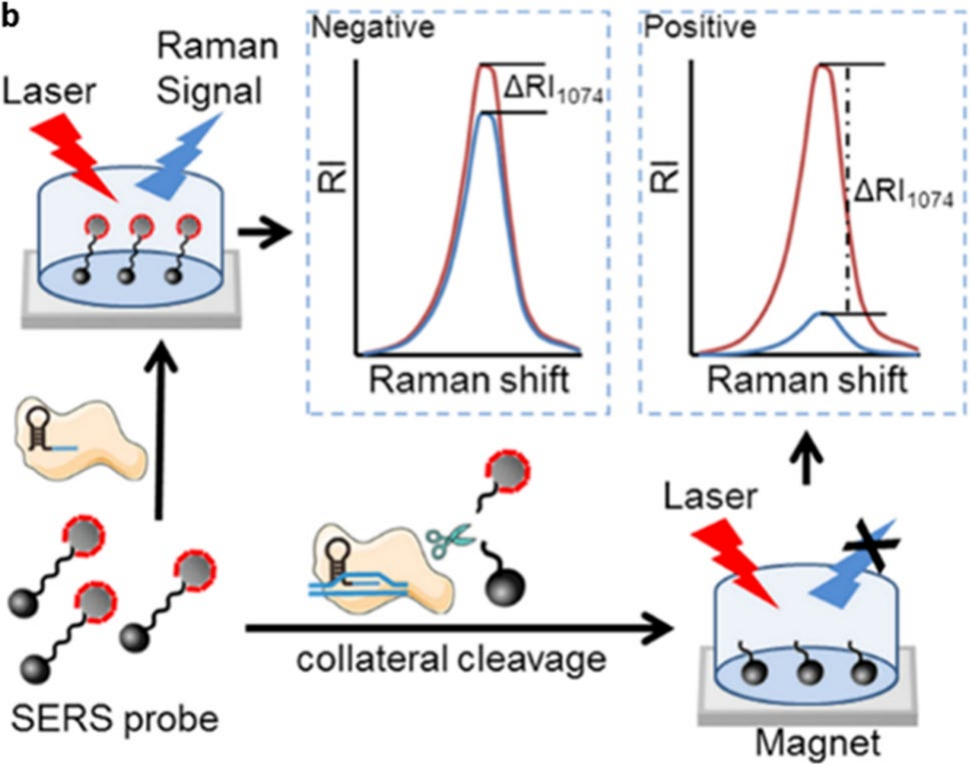
Raman detection via collateral cleavage of SERS probe (adapted from Liang et al. (2021))

In recent work, El-Said et al. have performed spectro-electrochemical detection of SARS-CoV-2 spike protein spiked in human serum samples (El-Said et al. 2022). Raman signal enhancement realized via the use of gold nanoparticles attached to a reduced porous graphene oxide modified substrate has resulted in obtaining a detection limit of 75 fmol L^−1^. A comparative evaluation of SARS-CoV-2 virions and purified RNA via Raman spectroscopy was reported in a recent proof-of-concept study, where a specific RNA marker band has been identified at 807 cm^−1^ (Wood et al. 2021). The potential of receptor binding domain (RBD) to be used as an effective target for SERS detection of the SARS-CoV-2 was also demonstrated recently (Awada et al. 2021). Ag/Au nanostructure fabricated on silicon rods via electroless etching and sputtering techniques was capable of detecting RBD protein up to 1 pM concentration in a short span (~ 3 s). In addition to detection, Raman spectroscopic tool has been also used to investigate the temperature response of spike protein in SARS-CoV-2 (Hernández-Arteaga et al. 2022). The spectral features of spike glycoprotein S1 started to show a significant intensity decline at 133 °C, whereas all the features were completely absent at 144 °C. SERS-based CRISPR (S-CRISPR) diagnostic test has been recently developed for detecting SARS-CoV-2 derived nucleic acids in RNA extracts collected from clinical specimens. This combined technique provides a reliable route for developing nucleic acid-based rapid point of care platforms, by eliminating the RNA amplification requirement in the traditional CRISPR/Cas-based tests. The various steps involved in viral identification from the nasopharyngeal swab samples were shown in Fig. 13 (Liang et al. 2021). The initial process comprised of the conventional RNA extraction from swab samples and further analysis was performed using a portable Raman plate reader. The presence of target DNA activates the Cas12a collateral cleavage, which separates the Raman tags from magnetic beads yielded a reduction in Raman signal intensity as shown in Fig. 13. In a recently published work, Choo et al. demonstrated a sensitive SERS technique for the detection of SARS-CoV-2 within 15 min using aptamer-based receptor on gold nano-popcorn substrate (Chen et al. 2021). Maiti et al. have demonstrated a proof of concept model for non-invasive COVID-19 detection by identifying the Raman signatures of saliva collected from infected patients at various stages. Using multivariate analytical tools with the developed Raman database, the model have been found effective in distinguishing healthy samples as well as different stages of disease recovery (Karunakaran et al. 2022). In another interesting work, the efficiency of COVID-19 vaccine has been verified from tear samples using SERS technique by synthesizing gold nanorods, nanoflowers, and nanospheres (Kim et al. 2022). The study conducted on limited sample size has been monitoring the spectral signatures of antibodies for identifying the vaccine status. The impact of the various nanostructure morphology on SERS-based viral detection have been investigated using bulk, dendritic, globular, and spiky silver nanostructures and found that dendrites have been showing efficacy in measurements in terms of sensitivity and repeatability (Li et al. 2022). Viral detection using SERS have been also reported by monitoring the changes in volatile organic compounds present in the exhaled breath samples within few minutes (Leong et al. 2022). In a recent work, SERS technique have been employed to evaluate the metabolic status in patients post COVID diagnosis (Chisanga et al. 2023). The study conducted in serum samples with a limited sample size (30 nos) has found variations in spectral signatures attributed to lipids and amino acids as possible markers in order to evaluate the disease recovery. Pack et al. have explored the high affinity of aptamers towards spike proteins for SERS-based detection with an LOD of 100 fg/mL (Park et al. 2023). The study employed aptamers coupled on a silver-based nanoforest surface prepared by sputtering technique for detecting recombinant spike protein by monitoring the Raman variations after protein-aptamer complex formation. Chiang et al. have developed a paper-based SERS substrate which can facilitate detection of SARS-CoV-2 variants which includes omicron, delta, and alpha (Yeh et al. 2023). The microplasma-engineered nanoassemblies fabricated on cellulose paper substrate were effective in enhancing the Raman signatures of spike proteins appearing at 2895 and 2946 cm^−1^.

Raman spectroscopy-based technologies reported for SARS-CoV-2 by various research groups are given in Table 2.

**Table 2:** Raman techniques for the detection of SARS-CoV-2 viruses

S. no	Method	Sample	LOD/sensitivity	Specificity	References
1	SERS	Water samples	–	–	Zhang et al. (2021a)
2	SERS	Contaminated water	80 copies/mL	–	Yang et al. (2021)
3	SERS	Serum samples	1.28 × 10^7^-fold dilution	100%	Liu et al. (2021)
4	SERS	COVID-19 antigen	4 × 10^−6^ µg/mL	–	Pramanik et al. (2021)
5	SERS	SARS-CoV-2 S protein solution	5 × 10^−9^ M	–	Peng et al. (2021)
6	SERS	Nasopharyngeal swabs	10 PFU/mL	–	Chen et al. (2021)
7	SERS	Saliva Serum Blood	Saliva—6.07 * 10^−6^ µg/mL Serum—7.60 * 10^−9^ µg/mL Blood—10^−7^ µg/mL	–	Zhang et al. (2021b)
8	SERS-based aptamer sensors	Nasopharyngeal swab	5.5 × 104 TCID50/mL	–	Zavyalova et al. (2021)
9	SERS	Purified SARS-CoV-2 spike (S) protein expressed in baculovirus insect cells	0.00889 µM	–	Sanchez et al. (2021)
10	SERS	Throat swabs	83.3%	92.5%	Huang et al. (2021)
11	SERS	SARS-CoV-2 RBD protein	10^−6^ µM	–	Awada et al. (2021)
12	SERS	Saliva	> 95%	> 95%	Carlomagno et al. (2021)
13	SERS	Blood serum	84.0%	95.0%	Goulart et al. (2021)
14	SERS	Nasopharyngeal swab	87.50%	100%	Liang et al. (2021)
15	Colorimetric/SERS/fluorescence	TE buffer	3.95 × 10^−7^ µM	–	Gao et al. (2021)
16	Raman spectroscopy	Spike glycol protein S1 of SARS-CoV-2	–	–	Hernández-Arteaga et al. (2022)
17	Raman spectroscopy, synchrotron infrared (IR) and AFM-IR	Saliva	93%	82%	Awada et al. (2021)
18	Square wave voltammetry (biosensor)	COVID-19 protein	3.95 × 10^−8^ µmol/L	–	El-Said et al. (2022)

## Prospects and challenges

This work has reviewed the potential of various forms of Raman spectroscopy in the diagnosis of viruses such as SARS-CoV-2. The narrow peak emission bands of the Raman spectra facilitate the multiplexed detection of virus biomarkers. The lack of interference from water absorption in Raman spectroscopy (as compared to standard infrared spectroscopy) makes it a favored technology for the analysis of body fluids. Utilization of plasmonic nanostructures to enhance the molecular fingerprinting capability of Raman spectroscopy provides advantages related to a lower LOD, broader linear range, higher sensitivity, and greater selectivity in viral diagnosis. Functionalization of plasmonic nanoparticles/nanostructures facilitates the use of target-specific or biomarker-specific SERS technique which is a prerequisite in clinical settings for viral studies. The SERS technique also lessens the requirement of sample volume and allows for simultaneous targeting of a variety of biomarkers.

The ability of SERS for virus detection is dictated by factors such as substrate design, substrate biocompatibility, laser condition, and analyte characteristics. Gold nanoparticles exhibit greater stability compared to silver or copper nanoparticles; however, the ability of silver to induce strong LSPR makes it a potential candidate for SERS substrate fabrication. The trade-off between stability and induction of LSPR upon electromagnetic wave incidence decides the optimum substrate for harvesting the SERS signal (Sitjar et al. 2021). Other 2D materials, such as graphene, have also been investigated as SERS substrates and indeed the application of unique multi-layered SERS substrate—(Cu2O-Au)-graphene–Au—for effective sensing applications has been suggested in recent literature. These proposed structures have been shown capable of providing high field enhancement (of the order of ~ 10^8^) at 1064 nm near-IR excitation, which can overcome problems related to visible light-induced fluorescence interference, photo-bleaching, and plasmonic heating, all of which impose limitations on the SERS effect (Nair & Murukeshan 2020). The orientation of analyte on the substrate surface also influences the SERS signal and hence has to be controlled by restricting the shape and size of nanoparticles. In the case of nanocavities, those virus particles having a size less than or comparable to the cavity can provide enhanced Raman signal. Xiao has come up with a “volume-enhanced Raman scattering (VERS)” approach which involves the fabrication of a substrate with volumetric nanoscale surface “hot spots.” The proposed substrate is made up of forming hollow nanocones at the bottom of microbowls (HNCMB). Unlike the conventional substrates, these VERS substrates facilitate an increase in the virus surface area which can enter the hotspots resulting in an enhanced collection of spectral information. These molecular imprinting-inspired substrates show superior single virus detection and reproducibility (Zhang et al. 2019). Virus particles attached to the top edges of nanorods can provide stronger signals since the major SERS effect is attributed to electromagnetic enhancement and the electromagnetic fields are stronger at sharp edges (Zhao et al. 2020). Spectral reproducibility is hampered by the non-uniform distribution and random aggregation of nanoparticles (Lu et al. 2022). The proper functionalization of affinity agents, such as antibodies and aptamers, on SERS substrates, can improve the specificity factor. Factors such as steric exclusion, random orientation on the substrate surface, and spectral interference tend to make aptamers a better candidate for functionalization of SERS substrate than antibodies (Tadesse et al. 2020).

The excitation wavelength and laser power play a critical role in the extraction of SERS signal with virus as analyte. Laser power considerations need to be balanced between extraction of a strong Raman signal without affecting substrate stability. Laser wavelength matching at (or near to) the plasmon resonance wavelength is desirable for the optimum SERS effect (Sitjar et al. 2021). In the resonance Raman spectroscopy technique, the laser wavelength is tuned to match with the electronic transition state of the molecule of interest but this requirement often demands a UV laser source for biomolecules. Virus integrity dictates the spectral features since the live virus, inactivated virus, enveloped virus, and non-enveloped virus are unique in their composition and activity. Mutations occurring in S-proteins (and other viral proteins) require regular updating of the SERS database. The investigation of clinical samples demands the rectification of Raman signature from other viruses present in the specimen, and so therefore multiplexed signal detection is mandatory. Advances in artificial intelligence and machine learning programs (that include multivariate statistical tools) allow for identification of receptor binding to viral spike proteins from SERS spectra. In view of the above, the setup for acquiring Raman signal from virus has to be designed to optimize signal strength by the considering the synergic effect all these factors.

The spatial resolution limitation associated with the traditional SERS approach can now be minimized by the use of TERS—which relies on the hot spot created at the apex of the SPM tip. Research on fabricating the TERS active sensor is still in progress, particularly with regard to the investigation of viruses. The TERS technique is more suitable for studying the mechanism of invasion/infection of viruses rather than their detection in patient samples. However, the effective utilization of the TERS technique demands significant user knowledge which currently limits the practical on-site applications via unskilled technicians, especially during pandemic crises (like COVID-19). Moreover, the associated low biological signal necessitates the fabrication of active probes that provide maximum plasmonic signal enhancement (Kumar et al. 2015). An alternate strategy involves using a higher integration time to achieve better signal strength; however, this often excludes recording the dynamics at a shorter duration of viral attack. Furthermore, the proximity of tips to the sample can lead to contamination or sample adsorption of the probe tip resulting in spurious results (Schmid et al. 2009). Though advances in micro and nanofabrication technologies have facilitated TERS tip fabrication, the fabrication of reproducible TERS tips is still a challenging task. Commonly adopted silver-based tips that provide high electric field enhancement are prone to oxidation or reaction with atmospheric sulfur consequently leading to deterioration of the plasmonic properties (Barrios et al. 2008). In addition, non-uniformity in sample surface features (e.g., roughness) can lead to fluctuating sample-probe distances and thus a varying TERS signal strength (Schmid et al. 2013).

In conclusion, although several features (such as rapid sampling, low LOD, excellent sensitivity and specificity, broad linear range) mark the SERS technique as particularly promising, its application in POC sensors for early viral detection is still at the beginning stages. The future potential of bifunctional nanoprobes for generating multiple spectral information (both Raman and fluorescence) from the complex biological samples should be explored further to enhance the sensitivity and specificity of virus detection (Xia et al. 2019). With the expectation of the integration of the SERS technique onto low-cost substrates like paper and polymers, the Raman spectroscopy technique is expected to provide fast, reliable, portable, low-cost sensors for the viral detection. Incorporation of the SERS technique into microfluidic platforms termed as “optofluidics” will facilitate high throughput sensing along with minimal sample volume requirement. Such highly reproducible, multiplexed, and molecule-specific SERS enhancement may enable a paradigm change in the POC technology for rapid detection of biomarkers from body fluids potentially becoming the preferred solution for rapid viral diagnosis in pandemics like COVID-19.

## Supplementary Information

Below is the link to the electronic supplementary material.

Supplementary file1 (PDF 278 KB)

## Data Availability

Not applicable (as this is a review article there is no raw data available).

## Code availability

Not applicable (as this is a review article there is no coding has been used).
