# Supplementary material for: Raman spectroscopy for viral diagnostics
Source: Biophys Rev. 2023 Apr 10;15(2):199–221. doi: 10.1007/s12551-023-01059-4 (PMC10088700; doi:10.1007/s12551-023-01059-4)
Supplement: Supplementary file 1 — Supplementary file1 (PDF 278 KB) [file 12551_2023_1059_MOESM1_ESM.pdf]

Supplementary Data

Supplementary Table. 1: Comparison of techniques reported for virus detection

S. No	Technique	Cost	Time	Highlights	Limitations	References
1	Plaque assay	Cost effective	1-2 weeks	High sensitivity	Time consuming, Operator dependent,	[1]
2	RT-PCR technique	High cost	2-4 hours	High sensitivity	High workload, Reagent shortage during epidemic	[2-4]
3	LAMP	Cost effective	30 minutes	Rapid, simple, high sensitivity and specificity	High chance of false positive results due to pH indicators	[5-7]
4	SHERLOCK	Cost effective	Rapid detection	No need of bulky instrumentation, sensitive and specific virus detection	Great chance of sample contamination	[6, 8, 9]
5	DETECTR	Cost effective	<40 minutes	Rapid, Easy to implement, Accurate	Need prior nucleic acid extraction and several physical procedures	[9]
6	FET based biosensors	Low cost	Few minutes	Highly sensitive and specific detection. No cross reactivity to MERS-CoV Antigen.	High quality graphene synthesis with identical parameter is a challenge	[10]
7	ELISA	Cost effective	Few hours	High-throughput detection, Easily	Professional skills are needed,	[11, 12]

				available, Helpful for disease prognosis and prevalence	Moderate accuracy	
8	Electrochemical biosensor	Low cost	10 to 30 seconds	Portable, High sensitivity, Used for bedside/on-site PoC diagnostics, minimum sample volume	Limited shelf life, Experts are needed, Interference to sample matrix	[13, 14]
9	CT Scan	High cost	30 minutes	Non-invasive	Low specificity, Expert is required for the operation,	[15, 16]

Supplementary Table 2. Raman spectroscopy for the detection of different types of viruses

**S. No.**	**Method**	**Sample**	**Disease/ Virus**	**LOD/Sensitivity**	**Specificity**	**Year/ References**
1	Ultraviolet Resonance Raman Spectroscopy	Buffer solution	Filamentous Virus fd	438 µg/ml	-	[17]
2	Ultraviolet Resonance Raman spectroscopy	Buffer solution	Pf1 (class II Filamentous Virus), fd virions	2052 µg/ml	-	[18]
3	Raman tweezers	Cell culture supernatants	Kaposi's Sarcoma-associated Herpes	-	-	[19]

			Virus (KSHV)			
4	SERS	MHN Buffer Solution	Respiratory Virus	100 PFU/ml	-	[20]
5	SERS	Serum-free DMEM	Respiratory Syncytial Virus (RSV)	-	-	[21]
6	Near Infrared Raman spectroscopy	Human blood serum	Hepatitis C	92%	88%	[22]
7	Raman spectroscopy	Human blood serum	Dengue Virus	-	-	[23]
8	SERS	Buffer	Coxsackievirus, Respiratory Syncytial Virus, E. coli, and B. cereus	-	-	[24]
9	TERS	Infected plant	Tobacco Mosaic Virus	-	-	[25]
10	SERS	-	Rotavirus	$\sim 10^4$ ffu/mL	100%	[26]
11	TERS	-	Avipoxvirus and Adenoassociated Virus	-	-	[27]
12	SERS	PBS sample	Rift Valley Fever Virus (RVFV) and West	WNV= 0.02 µM RVFV= 0.1 µM	-	[28]

			Nile Virus (WNV)			
13	Raman spectroscopy	Serum and whole blood	Dengue Virus	-	-	[29]
14	SERS	Tris buffer	AIV PB1-F2 Protein	~0.01 μM	-	[30]
15	Raman spectroscopy	Human embryonic kidney epithelial (HEK293) cells	Adenovirus	-	-	[31]
16	Raman spectroscopy and advanced statistical methods	African green monkey kidney (Vero) cells	Herpes Simplex Virus type 1 (HSV-1)	100%	-	[32]
17	SERS	Human blood	Hepatitis B Virus	0.01 IU/mL	-	[33]
18	SERS	DMEM	Human Immunodeficienc Virus (HIV-1 Virus)	3.5×10^{-8} μg/mL	-	[34]
19	SERS	PBS	Influenza Virus (H_1N_1)	-	-	[35]
20	TERS	-	Varicella-zoster Virus (VZV) and Porcine	-	-	[36]

			Teschovirus (PTV)			
21	SERS	PBS sample	Enterovirus 71 protein	10^5 PFU/mL	-	[37]
22	SERS	Saliva	Salivary NS1	96.9%	100%	[38]
23	SERS-LFIA	Serum sample	Zika and Dengue Viruses	0.00072 µg/mL of ZIKV NS1 and 0.00767 µg/mL of DENV NS1	-	[39]
24	SERS	Human serum	H5N1	7.4×10^{-5} µg/mL		[40]
25	Raman spectroscopy	human embryonic kidney 293 (HEK293) cells	Adenovirus	-	-	[41]
26	SERS	PBS sample	Zika virus, NS1 Antigen	0.01 µg/mL	-	[42]
27	SERS	Serum	Hepatitis B Surface Antigen (HBsAg)	5×10^{-8} µg/mL	-	[43]
28	SERS	Serum	Hepatitis B	91.4%	83%	[44]
29	SERS-based LFIA	Human blood Serum	Influenza A H1N1 Virus and Human Adenovirus (HAdV)	H1N1=50 PFU/mL HAdV= 10 PFU/mL,	-	[45]

30	TERS	-	Influenza Virus and Picornavirus	-	-	[46]
31	AFM-IR, TERS	TNE Buffer	Herpes Simplex Type 1 Virus(HSV-1)	-	-	[47]
32	SERS	Blood serum	Hepatitis C Virus	-	-	[48]
33	SERS	Nasopharyngeal swabs	Rhinovirus, Influenza Virus, and Parainfluenza Viruses	10^2 $EID_{50}/\mu L$ (50% egg infective dose per microliter)	90%	[49]
34	SERS	HeLa cell lysate	Influenza A Virus	-	-	[50]
35	Raman spectroscopy	Human glial cells	Epstein-Barr Virus	-	-	[51]
36	SERS	Peripheral blood mononuclear cells (PBMCs)	Human Immunodeficiency Virus (HIV-1)	10^2 to 2×10^5 copies/mL	84.8%	[52]
37	Raman spectroscopy	Human saliva	RNA virus	92.5%	88.8%	[53]
38	SERS imaging-based aptasensor	Virus titre solution	Influenza Virus A (H1N1, H3N2)	97 PFU/mL	-	[54]
39	SERS	Blood serum	Dengue	-	-	[55]
40	SERS	Blood	Hepatitis C Virus	-	-	[56]

41	Raman spectroscopy	Serum	Hepatitis	-	94.54 %	[57]
42	SERS Immunochromatographic Assay (ICA)	Clinical feces samples	Rotavirus	8×10^{-6} µg/mL	-	[58]
43	SERS	PCR products	Hepatitis B	89%	98%	[59]
44	Raman Spectroscopy	Blood plasma	Hepatitis B	100%	99.25 %	[60]
45	Raman	Cell cultures	Porcine Parvovirus	95.55% to 97.77%	100%	[61]

References

[1] G.A. Storch, Diagnostic virology, Clinical infectious diseases, 31 (2000) 739-751.

[2] M. Espy, J. Uhl, L. Sloan, S. Buckwalter, M. Jones, E. Vetter, J. Yao, N. Wengenack, J. Rosenblatt, F. Cockerill III, Real-time PCR in clinical microbiology: applications for routine laboratory testing, Clinical microbiology reviews, 19 (2006) 165-256.

[3] J.B. Aguilar, J.S. Faust, L.M. Westafer, J.B. Gutierrez, Investigating the impact of asymptomatic carriers on COVID-19 transmission, MedRxiv, (2020).

[4] J.N. Rauch, E. Valois, S.C. Solley, F. Braig, R.S. Lach, M. Audouard, J.C. Ponce-Rojas, M.S. Costello, N.J. Baxter, K.S. Kosik, A scalable, easy-to-deploy protocol for Cas13-based detection of SARS-CoV-2 genetic material, Journal of clinical microbiology, 59 (2021) e02402-02420.

[5] W. Feng, A.M. Newbigging, C. Le, B. Pang, H. Peng, Y. Cao, J. Wu, G. Abbas, J. Song, D.-B. Wang, Molecular diagnosis of COVID-19: challenges and research needs, Analytical chemistry, 92 (2020) 10196-10209.

[6] N. Taleghani, F. Taghipour, Diagnosis of COVID-19 for controlling the pandemic: A review of the state-of-the-art, Biosensors and Bioelectronics, (2020) 112830.

[7] M. Jiang, W. Pan, A. Arasthfer, W. Fang, L. Ling, H. Fang, F. Daneshnia, J. Yu, W. Liao, H. Pei, Development and validation of a rapid, single-step reverse transcriptase loop-mediated isothermal amplification (RT-LAMP) system potentially to be used for reliable and high-throughput screening of COVID-19, Frontiers in cellular and infection microbiology, 10 (2020) 331.

[8] F. Zhang, O.O. Abudayyeh, J.S. Gootenberg, A protocol for detection of COVID-19 using CRISPR diagnostics, A protocol for detection of COVID-19 using CRISPR diagnostics, 8 (2020).

[9] J.P. Broughton, X. Deng, G. Yu, C.L. Fasching, V. Servellita, J. Singh, X. Miao, J.A. Streithorst, A. Granados, A. Sotomayor-Gonzalez, CRISPR–Cas12-based detection of SARS-CoV-2, Nature biotechnology, 38 (2020) 870-874.

[10] G. Seo, G. Lee, M.J. Kim, S.-H. Baek, M. Choi, K.B. Ku, C.-S. Lee, S. Jun, D. Park, H.G. Kim, Rapid detection of COVID-19 causative virus (SARS-CoV-2) in human nasopharyngeal swab specimens using field-effect transistor-based biosensor, ACS nano, 14 (2020) 5135-5142.
[11] S.K. Lau, P.C. Woo, B.H. Wong, H.-W. Tsoi, G.K. Woo, R.W. Poon, K.-H. Chan, W.I. Wei, J.M. Peiris, K.-Y. Yuen, Detection of severe acute respiratory syndrome (SARS) coronavirus nucleocapsid protein in SARS patients by enzyme-linked immunosorbent assay, Journal of clinical microbiology, 42 (2004) 2884-2889.
[12] A.E. Dhamad, M.A.A. Rhida, COVID-19: molecular and serological detection methods, PeerJ, 8 (2020) e10180.
[13] S. Menon, M.R. Mathew, S. Sam, K. Keerthi, K.G. Kumar, Recent advances and challenges in electrochemical biosensors for emerging and re-emerging infectious diseases, Journal of Electroanalytical Chemistry, (2020) 114596.
[14] S. Mahari, A. Roberts, D. Shahdeo, S. Gandhi, eCovSens-ultrasensitive novel in-house built printed circuit board based electrochemical device for rapid detection of nCovid-19 antigen, a spike protein domain 1 of SARS-CoV-2, BioRxiv, (2020).
[15] D. Wu, T. Wu, Q. Liu, Z. Yang, The SARS-CoV-2 outbreak: what we know, International Journal of Infectious Diseases, 94 (2020) 44-48.
[16] T. Ai, Z. Yang, H. Hou, C. Zhan, C. Chen, W. Lv, Q. Tao, Z. Sun, L. Xia, Correlation of chest CT and RT-PCR testing for coronavirus disease 2019 (COVID-19) in China: a report of 1014 cases, Radiology, 296 (2020) E32-E40.
[17] Z.Q. Wen, S.A. Overman, G.J. Thomas, Structure and interactions of the single-stranded DNA genome of filamentous virus fd: investigation by ultraviolet resonance Raman spectroscopy, Biochemistry, 36 (1997) 7810-7820.
[18] Z.Q. Wen, A. Armstrong, G.J. Thomas, Demonstration by ultraviolet resonance Raman spectroscopy of differences in DNA organization and interactions in filamentous viruses Pf1 and fd, Biochemistry, 38 (1999) 3148-3156.
[19] K.E. Hamden, B.A. Bryan, P.W. Ford, C. Xie, Y.-Q. Li, S.M. Akula, Spectroscopic analysis of Kaposi's sarcoma-associated herpesvirus infected cells by Raman tweezers, Journal of virological methods, 129 (2005) 145-151.
[20] S. Shanmukh, L. Jones, J. Driskell, Y. Zhao, R. Dluhy, R.A. Tripp, Rapid and sensitive detection of respiratory virus molecular signatures using a silver nanorod array SERS substrate, Nano letters, 6 (2006) 2630-2636.
[21] S. Shanmukh, L. Jones, Y.-P. Zhao, J. Driskell, R. Tripp, R. Dluhy, Identification and classification of respiratory syncytial virus (RSV) strains by surface-enhanced Raman spectroscopy and multivariate statistical techniques, Analytical and bioanalytical chemistry, 390 (2008) 1551-1555.
[22] J. Saade, M.T.T. Pacheco, M.R. Rodrigues, L. Silveira Jr, Identification of hepatitis C in human blood serum by near-infrared Raman spectroscopy, Spectroscopy, 22 (2008) 387-395.
[23] K. Naseer, A. Amin, M. Saleem, J. Qazi, Raman spectroscopy based differentiation of typhoid and dengue fever in infected human sera, Spectrochimica Acta Part A: Molecular and Biomolecular Spectroscopy, 206 (2019) 197-201.
[24] M. Demirel, P. Kao, N. Malvadkar, H. Wang, X. Gong, M. Poss, D. Allara, Bio-organism sensing via surface enhanced Raman spectroscopy on controlled metal/polymer nanostructured substrates, Biointerphases, 4 (2009) 35-41.
[25] D. Cialla, T. Deckert-Gaudig, C. Budich, M. Laue, R. Möller, D. Naumann, V. Deckert, J. Popp, Raman to the limit: tip-enhanced Raman spectroscopic investigations of a single tobacco

mosaic virus, Journal of Raman Spectroscopy: An International Journal for Original Work in all Aspects of Raman Spectroscopy, Including Higher Order Processes, and also Brillouin and Rayleigh Scattering, 40 (2009) 240-243.
[26] J.D. Driskell, Y. Zhu, C.D. Kirkwood, Y. Zhao, R.A. Dluhy, R.A. Tripp, Rapid and sensitive detection of rotavirus molecular signatures using surface enhanced Raman spectroscopy, PloS one, 5 (2010) e10222.
[27] P. Hermann, A. Hermelink, V. Lausch, G. Holland, L. Möller, N. Bannert, D. Naumann, Evaluation of tip-enhanced Raman spectroscopy for characterizing different virus strains, Analyst, 136 (2011) 1148-1152.
[28] H. Zhang, M.H. Harpster, W.C. Wilson, P.A. Johnson, Surface-enhanced Raman scattering detection of DNAs derived from virus genomes using Au-coated paramagnetic nanoparticles, Langmuir, 28 (2012) 4030-4037.
[29] A. Rehman, S. Anwar, S. Firdous, M. Ahmed, R. Rasheed, M. Nawaz, Dengue blood analysis by Raman spectroscopy, Laser Physics, 22 (2012) 1085-1089.
[30] P. Negri, R.A. Dluhy, Detection of genetic markers related to high pathogenicity in influenza by SERS, Analyst, 138 (2013) 4877-4884.
[31] K. Moor, K. Ohtani, D. Myrzakozha, O. Zhanserkenova, B.B. Andriana, H. Sato, Noninvasive and label-free determination of virus infected cells by Raman spectroscopy, Journal of biomedical optics, 19 (2014) 067003.
[32] A. Salman, E. Shufan, L. Zeiri, M. Huleihel, Characterization and detection of Vero cells infected with Herpes Simplex Virus type 1 using Raman spectroscopy and advanced statistical methods, Methods, 68 (2014) 364-370.
[33] A. Kamińska, E. Witkowska, K. Winkler, I. Dzięcielewski, J.L. Weyher, J. Waluk, Detection of Hepatitis B virus antigen from human blood: SERS immunoassay in a microfluidic system, Biosensors and bioelectronics, 66 (2015) 461-467.
[34] J.-H. Lee, B.-C. Kim, O. Byeung-Keun, J.-W. Choi, Rapid and sensitive determination of HIV-1 virus based on surface enhanced raman spectroscopy, Journal of biomedical nanotechnology, 11 (2015) 2223-2230.
[35] J.-y. Lim, J.-s. Nam, S.-e. Yang, H. Shin, Y.-h. Jang, G.-U. Bae, T. Kang, K.-i. Lim, Y. Choi, Identification of newly emerging influenza viruses by surface-enhanced Raman spectroscopy, Analytical chemistry, 87 (2015) 11652-11659.
[36] K. Olschewski, E. Kämmer, S. Stöckel, T. Bocklitz, T. Deckert-Gaudig, R. Zell, D. Cialla-May, K. Weber, V. Deckert, J. Popp, A manual and an automatic TERS based virus discrimination, Nanoscale, 7 (2015) 4545-4552.
[37] M. Reyes, M. Piotrowski, S.K. Ang, J. Chan, S. He, J.J.H. Chu, J.C.Y. Kah, Exploiting the anti-aggregation of gold nanostars for rapid detection of hand, foot, and mouth disease causing enterovirus 71 using surface-enhanced Raman spectroscopy, Analytical chemistry, 89 (2017) 5373-5381.
[38] N. Othman, K.Y. Lee, A. Radzol, W. Mansor, N. Ramlan, Linear discriminant analysis for detection of salivary NS1 from SERS spectra, TENCON 2017-2017 IEEE Region 10 Conference, IEEE, 2017, pp. 2876-2879.
[39] M. Sánchez-Purrà, M. Carré-Camps, H. de Puig, I. Bosch, L. Gehrke, K. Hamad-Schifferli, Surface-enhanced Raman spectroscopy-based sandwich immunoassays for multiplexed detection of Zika and Dengue viral biomarkers, ACS infectious diseases, 3 (2017) 767-776.

[40] Y. Wang, Q. Ruan, Z.-C. Lei, S.-C. Lin, Z. Zhu, L. Zhou, C. Yang, Highly sensitive and automated surface enhanced Raman scattering-based immunoassay for H5N1 detection with digital microfluidics, Analytical chemistry, 90 (2018) 5224-5231.
[41] K. Moor, Y. Terada, A. Taketani, M. Hiroko, K. Ohtani, H. Sato, Early detection of virus infection in live human cells using Raman spectroscopy, Journal of biomedical optics, 23 (2018) 097001.
[42] S.A. Camacho, R.G. Sobral-Filho, P.H.B. Aoki, C.J.L. Constantino, A.G. Brolo, Zika immunoassay based on surface-enhanced raman scattering nanoprobes, ACS sensors, 3 (2018) 587-594.
[43] M. Liu, C. Zheng, M. Cui, X. Zhang, D.-P. Yang, X. Wang, D. Cui, Graphene oxide wrapped with gold nanorods as a tag in a SERS based immunoassay for the hepatitis B surface antigen, Microchimica Acta, 185 (2018) 1-8.
[44] Y. Lu, Y. Lin, Z. Zheng, X. Tang, J. Lin, X. Liu, M. Liu, G. Chen, S. Qiu, T. Zhou, Label free hepatitis B detection based on serum derivative surface enhanced Raman spectroscopy combined with multivariate analysis, Biomedical optics express, 9 (2018) 4755-4766.
[45] C. Wang, C. Wang, X. Wang, K. Wang, Y. Zhu, Z. Rong, W. Wang, R. Xiao, S. Wang, Magnetic SERS strip for sensitive and simultaneous detection of respiratory viruses, ACS applied materials & interfaces, 11 (2019) 19495-19505.
[46] V. Deckert, T. Deckert-Gaudig, D. Cialla-May, J. Popp, R. Zell, S. Deinhard-Emmer, A.V. Sokolov, Z. Yi, M.O. Scully, Laser spectroscopic technique for direct identification of a single virus I: FASTER CARS, Proceedings of the National Academy of Sciences, 117 (2020) 27820-27824.
[47] T. Dou, Z. Li, J. Zhang, A. Evilevitch, D. Kurouski, Nanoscale structural characterization of individual viral particles using atomic force microscopy infrared spectroscopy (AFM-IR) and tip-enhanced raman spectroscopy (TERS), Analytical Chemistry, 92 (2020) 11297-11304.
[48] M. Kashif, M.I. Majeed, M.A. Hanif, A. ur Rehman, Surface Enhanced Raman Spectroscopy of the serum samples for the diagnosis of Hepatitis C and prediction of the viral loads, Spectrochimica Acta Part A: Molecular and Biomolecular Spectroscopy, 242 (2020) 118729.
[49] Y.-T. Yeh, K. Gulino, Y. Zhang, A. Sabestien, T.-W. Chou, B. Zhou, Z. Lin, I. Albert, H. Lu, V. Swaminathan, A rapid and label-free platform for virus capture and identification from clinical samples, Proceedings of the National Academy of Sciences, 117 (2020) 895-901.
[50] K. Dardir, H. Wang, B.E. Martin, M. Atzampou, C.B. Brooke, L. Fabris, SERS nanoprobe for intracellular monitoring of viral mutations, The Journal of Physical Chemistry C, 124 (2020) 3211-3217.
[51] D. Tiwari, S. Jakhmola, D.K. Pathak, R. Kumar, H.C. Jha, Temporal In Vitro Raman Spectroscopy for Monitoring Replication Kinetics of Epstein–Barr Virus Infection in Glial Cells, ACS omega, 5 (2020) 29547-29560.
[52] S. Yadav, S. Senapati, D. Desai, S. Gahlaut, S. Kulkarni, J. Singh, Portable and sensitive Ag nanorods based SERS platform for rapid HIV-1 detection and tropism determination, Colloids and Surfaces B: Biointerfaces, 198 (2021) 111477.
[53] S. Desai, S.V. Mishra, A. Joshi, D. Sarkar, A. Hole, R. Mishra, S. Dutt, M.K. Chilakapati, S. Gupta, A. Dutt, Raman spectroscopy-based detection of RNA viruses in saliva: A preliminary report, Journal of Biophotonics, 13 (2020) e202000189.
[54] H. Chen, S.-G. Park, N. Choi, J.-I. Moon, H. Dang, A. Das, S. Lee, D.-G. Kim, L. Chen, J. Choo, SERS imaging-based aptasensor for ultrasensitive and reproducible detection of influenza virus A, Biosensors and Bioelectronics, 167 (2020) 112496.

[55] S. Gahlaut, D. Savargaonkar, C. Sharan, S. Yadav, P. Mishra, J. Singh, SERS platform for dengue diagnosis from clinical samples employing a hand held Raman spectrometer, Analytical chemistry, 92 (2020) 2527-2534.
[56] S. Nasir, M.I. Majeed, H. Nawaz, N. Rashid, S. Ali, S. Farooq, M. Kashif, S. Rafiq, S. Bano, M.N. Ashraf, Surface enhanced Raman spectroscopy of RNA samples extracted from blood of hepatitis C patients for quantification of viral loads, Photodiagnosis and Photodynamic Therapy, 33 (2021) 102152.
[57] Y. Zhao, S. Tian, L. Yu, Z. Zhang, W. Zhang, Analysis and Classification of Hepatitis Infections Using Raman Spectroscopy and Multiscale Convolutional Neural Networks, Journal of Applied Spectroscopy, 88 (2021) 441-451.
[58] Y. Zhang, G. Wu, J. Wei, Y. Ding, Y. Wei, Q. Liu, H. Chen, Rapid and sensitive detection of rotavirus by surface-enhanced Raman scattering immunochromatography, Microchimica Acta, 188 (2021) 1-10.
[59] F. Batool, H. Nawaz, M.I. Majeed, N. Rashid, S. Bashir, S. Akbar, M. Abubakar, S. Ahmad, M.N. Ashraf, S. Ali, SERS-based viral load quantification of hepatitis B virus from PCR products, Spectrochimica Acta Part A: Molecular and Biomolecular Spectroscopy, 255 (2021) 119722.
[60] S. Ali, M. Hassan, M. Saleem, S.F. Tahir, Deep transfer learning based hepatitis B virus diagnosis using spectroscopic images, International Journal of Imaging Systems and Technology, 31 (2021) 94-105.
[61] I.C. Gogone, G.H. Ferreira, D. Gava, R. Schaefer, F.F. de Paula-Lopes, R.d.A. Rocha, F.R. de Barros, Applicability of Raman spectroscopy on porcine parvovirus and porcine circovirus type 2 detection, Spectrochimica Acta Part A: Molecular and Biomolecular Spectroscopy, 249 (2021) 119336.
